# Environmental Enrichment Attenuates Repetitive Behavior and Alters the Functional Connectivity of Pain and Sensory Pathways in C58 Mice

**DOI:** 10.3390/cells13231933

**Published:** 2024-11-21

**Authors:** Anna L. Farmer, Marcelo Febo, Bradley J. Wilkes, Mark H. Lewis

**Affiliations:** 1 Department of Psychology, University of Florida, Gainesville, FL 32603, USA; marklewis@ufl.edu; 2 Department of Psychiatry, University of Florida, Gainesville, FL 32611, USA; febo@ufl.edu; 3 Department of Applied Physiology and Kinesiology, University of Florida, Gainesville, FL 32608, USA; bwilkes@ufl.edu

**Keywords:** autism spectrum disorder, restricted repetitive behavior, environment, functional magnetic resonance imaging, fMRI, pain, somatosensory

## Abstract

Restricted repetitive behaviors (RRB) encompass a variety of inflexible behaviors, which are diagnostic for autism spectrum disorder (ASD). Despite being requisite diagnostic criteria, the neurocircuitry of these behaviors remains poorly understood, limiting treatment development. Studies in translational animal models show environmental enrichment (EE) reduces the expression of RRB, although the underlying mechanisms are largely unknown. This study used functional magnetic resonance imaging to identify functional connectivity alterations associated with RRB and its attenuation by EE in C58 mice, an animal model of RRB. Extensive differences were observed between C58 mice and C57BL/6 control mice. Higher RRB was associated with altered connectivity between the somatosensory network and reticular thalamic nucleus and between striatal and sensory processing regions. Animals housed in EE displayed increased connectivity between the somatosensory network and the anterior pretectal nucleus and hippocampus, as well as reduced connectivity between the visual network and area prostriata. These results suggest aberrant sensory perception is associated with RRB in C58 mice. EE may reduce RRB by altering functional connectivity in pain and visual networks. This study raises questions about the role of sensory processing and pain in RRB development and identifies new potential intervention targets.

## 1. Introduction

Restricted repetitive behaviors (RRBs) encompass a wide variety of inflexible cognitive and motor behaviors [1,2]. Although these behaviors are diagnostic for autism spectrum disorder (ASD), their neurobiological basis remains poorly understood [3,4]. This poor understanding of the underlying neural circuitry has hampered the development of effective treatments [4,5]. Interestingly, numerous studies have demonstrated that environmental enrichment (EE) dramatically reduces RRB in animal disease models and zoological settings [6]. EE is known to induce widespread neurobiological alterations [7,8,9]; however, few studies have investigated the neurobiological mechanisms underlying EE’s reduction of RRB. Identifying these mechanisms holds promise for both furthering the understanding of the neural basis of RRB, as well as the identification of targets for the development of RRB treatments.

Resting state functional magnetic resonance imaging (fMRI) is a non-invasive and powerful tool for identifying neurocircuitry alterations associated with neuropathological states [10]. Few fMRI studies have examined functional connectivity differences associated with RRB. In a human fMRI study, Delmonte et al. [11] discovered that increased functional connectivity between the right middle frontal gyrus and caudate was correlated with higher RRB in adolescent and young adult males. Uddin et al. [12] found an association between increased intra-network functional connectivity within the salience network and higher RRB in children with ASD but did not examine associations with other functional networks. In contrast, Weng et al. [13] observed an association between hypoconnectivity within the default mode network and higher RRB scores on the Autism Diagnostic Interview—Revised assessment in adolescents. In an fMRI study of an autism animal model, Sforazzini et al. [14] observed widespread functional connectivity differences in BTBR mice compared to control mice but did not examine the relationship between functional connectivity differences and RRB behavior. Thus, multiple brain regions and networks have been implicated in RRB, but the current literature is too sparse to provide a clear consensus on the underlying functional changes. To our knowledge, no prior studies have used fMRI methods to examine the effects of EE on RRB.

C58 mice are an inbred mouse strain that naturally and spontaneously engage in frequent bouts of repetitive motor behaviors [15,16] and cognitive inflexibility during reversal learning tasks [17,18]. RRB in C58 mice can be attenuated by exposure to environmental enrichment [15,16,18], although investigations into the underlying mechanisms have been limited. Lewis et al. [15] observed increased dendritic spine density and gene expression of genes associated with synaptic plasticity and glutamatergic receptors in the subthalamic nucleus of C58 mice exposed to EE for six weeks post-weaning. Additional studies in deer mice suggest EE reduces RRB by increasing neuronal activity and synaptic plasticity in the motor cortex and other basal ganglia regions [19,20,21,22,23]. Turner et al. [23] also observed increased neuronal activity in the frontal cortex, hippocampus, and thalamus of deer mice exposed to an enriched environment for 60 days. Understanding the neural mechanisms responsible for the spontaneous RRB in C58 mice and its attenuation by EE in these mice may provide useful insights into RRB in autism and other neurodevelopmental disorders.

We previously used diffusion tensor imaging (DTI) to examine how EE alters brain microstructure to attenuate the development of RRB in C58 mice, an animal model of RRB [24]. In that study, we examined brain microstructure differences between C58 mice and C57BL/6J (C57) mice, a closely related control strain that does not typically engage in RRB. We found that C58 mice exhibited widespread microstructural differences in gray and white matter from C57 mice when both are housed under standard laboratory conditions. We also demonstrated that EE extensively altered gray matter microstructure in juvenile C58 mice and that RRB attenuation by EE was associated with increases in fractional anisotropy and axial diffusivity in the cerebellum, medial entorhinal cortex, and sensory processing regions. In this current paper, we examined fMRI data from these same animals in conjunction with the RRB measures reported in Farmer et al. [24] to determine if functional connectivity alterations associated with RRB and EE mirror the structural alterations we previously observed with DTI. In the present study, we examine functional connectivity differences between C58 and C57 mice to identify alterations underlying strain differences in RRB expression. We also investigate the relationship between resting state network connectivity and repetitive motor scores and compare the effects of housing (EE versus standard lab cages) on brain functional connectivity. Lastly, we demonstrate how the relationship between RRB and functional connectivity changes during development in C58 mice by examining two age cohorts: juvenile animals in which RRB has not yet fully developed and an older cohort of young adult animals with fully developed RRB.

## 2. Materials and Methods

### 2.1. Animal Housing

Animal housing was as described in [24]. Briefly, C58/J and C57BL/6J inbred mice were acquired from the Jackson laboratory (Bar Harbor, ME, USA) and bred and housed in a colony at the University of Florida. All mice were within 5 generations of founding colony pairs. We evaluated two different age cohorts. In adult mice, we performed behavioral measures and MRI scans at six weeks post-weaning, a time when repetitive motor behaviors are well established in C58 mice. We also evaluated a younger cohort of juvenile mice in which behavioral and MRI measures were conducted at three weeks post-weaning, a time when C58 mice have not yet reached adult levels of repetitive behavior [16]. Both age cohorts were weaned on postnatal day 21 and assigned to either standard housing (SH) or environmental enrichment housing (EE), balancing for sex and litter effects.

All animals were housed socially with three to six same-sex mice per cage with continuous access to food and water. SH housing consisted of plastic cages (29 × 18 × 13 cm) with bedding and nestlets. EE housing consisted of a large dog kennel (122 × 81 × 89 cm) with multiple levels and bedding, a plastic shelter, running wheels to provide opportunities for physical exercise, nestlets for next construction, Habitrail tubes, and four toys per level that were changed every two weeks to provide sensory stimulation. EE kennels had scattered bird seed (2 oz/week) to allow foraging behavior, whereas bird seed (2 oz/week) for the SH group was provided in a corner of the cage. All animals were kept in the same room, which had a 12 h light/dark cycle and a temperature of 70–75 °F. At the start of behavioral assessment, EE animals were temporarily transferred to a plastic cage (29 × 18 × 13 cm) with bedding, nestlets, a small running wheel, plastic hut, a Habitrail tube, and a single toy to enable easier capture. To ensure the ethical treatment of animals, all animal care and procedures were approved by the University of Florida Institutional Animal Care and Use Committee and conducted in compliance with the US National Research Council’s Guide for the Care and Use of Laboratory Animals, as well as the US Public Health Service’s Policy on Humane Care and Use of Laboratory Animals.

### 2.2. Behavioral Assessment and Analyses

Repetitive motor behavior assessments were performed as previously described by [24]. Briefly, these were conducted overnight throughout the dark cycle using photobeam arrays to quantify vertical movements (hindlimb jumping and backward somersaulting). During behavioral assessment, mice were temporarily housed in individual test cages with continuous access to food and water. Automated counts were verified in a subsample of video recordings by trained observers.

### 2.3. Magnetic Resonance Imaging

#### 2.3.1. Image Acquisition

MRI scans were conducted within 1 week of behavior assessments. Anesthesia was induced for MRI scans using 2.5–3% isoflurane followed by an intraperitoneal injection of 0.1 mg/kg dexmedetomidine (Dexdomitor, Zoetis, Parsippany, NJ 07054, USA; 1 mL/kg volume). Mice were scanned while sedated using a combination of 0.5% isoflurane (0.4 L/min mixed with medical grade air) via a nosecone and a constant subcutaneous infusion of dexmedetomidine (0.05 mg/kg/mL over 1 h using a PHD-Ultra microinfusion pump, Harvard Apparatus, Holliston, MA, USA). Functional MRI scans were collected at least 40 min after the intraperitoneal injection. Respiratory rates were monitored continuously using a respiratory pad placed under the abdomen, and body temperature was maintained at 36–37 °C using a warm water recirculation system (SA Instruments, Inc., New York, NY, USA).

MRI imaging was conducted at the Advanced Magnetic Resonance Imaging and Spectroscopy (AMRIS) facility at the University of Florida on an 11.1 T scanner (Magnex Scientific Ltd., Oxford, UK) with Resonance Research Inc. (Billerica, MA, USA) gradients (RRI BFG-240/120-S6, maximum gradient strength of 1000 mT/m at 325 Amps and a 200 µs risetime) and an Advance III Bruker Paravision 6.01 console (Bruker BioSpin, Billerica, MA, USA). A custom-made 2 cm × 2.5 cm quadrature surface transmit/receive coil (470.7 MHz) was placed on the mouse’s head during scans. For each mouse, we acquired a high-resolution T2 weighted anatomical scan and a functional magnetic resonance imaging (fMRI) scan. The T2-weighted Turbo Rapid Acquisition with Refocused Echoes (TurboRARE) sequence had an effective echo time (TE) of 41 ms, repetition time (TR) of 4 s, 15 mm × 15 mm × 12.6 mm field of view with a data matrix of 256 × 256 with 14 interleaved slices, 58.59 μm × 58.59 μm × 900 μm resolution, RARE factor of 16, and 12 averages. Functional images were collected using a single-shot spin-echo echo planar imaging (SE-EPI) sequence with the following parameters: TE = 16 ms, TR = 1.5 s, 600 repetitions, FOV = 15 × 15 mm × 12.6 mm, resolution of 234.4 μm × 312.5 μm × 900 μm, and a data matrix of 64 × 48 with 14 interleaved ascending coronal slices in the same space as the T2 anatomical. Two single-repetition SE-EPI scans with phase encode gradient lobes collected along the positive and negative gradient direction were also collected for distortion correction. Scans covered the entire brain from the olfactory bulb to the spinal cord.

#### 2.3.2. Image Preprocessing

Preprocessing of MRI scans was similar to that in [25], using software tools in Analysis of Functional NeuroImages (AFNI) [26], FMRIB Software Library (FSL) [27], and Advanced Normalization Tools (ANTs) [28]. Brain masks were created for the anatomical and fMRI scans in MATLAB using Three-Dimensional Pulsed Coupled Neural Networks (PCNN3D) [29] followed by manual correction in ITK-SNAP [30]. fMRI preprocessing consisted of the application of FSL topup to remove susceptibility-related local magnetic field distortions, 3dDespike in AFNI to remove spikes in the time series, AFNI’s 3dvolreg to correct for motion and linear drift, AFNI’s 3dTproject to remove low frequency signals with a highpass filter of 0.0009 Hz, and the removal of additional noise components using an independent component analysis in FSL Melodic and FSL regfilt. All images were then reoriented to LPI orientation followed by N4BiasFieldCorrection in ANTs of the anatomical scan to correct for field inhomogeneities. The anatomical scan was then linearly registered to a twice down-sampled mouse brain template (Allen Mouse Brain Atlas) [31] using FSL’s linear registration tool (flirt) followed by nonlinear warping using ANTs. The anatomical registration matrices were then applied to the fMRI scan to align it with the mouse brain template.

### 2.4. Functional Connectivity Analysis

Following preprocessing, fMRI scans were included in an independent component analysis (ICA) in FSL MELODIC [32] to identify 20 independent components representing resting state networks. In the older mouse cohort, all comparisons were based on an ICA that included 78 mice from both strains, housing types, and sexes (Table 1). Separate ICAs were conducted for each age cohort. In the younger cohort, which only included C58 mice, a 20-component ICA was conducted with 18 C58 mice (Table 1). Separate two-tailed *t*-tests were conducted to determine functional connectivity differences in resting state networks between mouse strains and housing conditions. For both age cohorts, a correlation analysis was conducted with repetitive motor scores to identify brain regions with significant correlations between RRB and functional connectivity differences in resting state networks. All MRI statistical analyses were conducted in FSL dual regression [33] with 5000 permutations and threshold-free cluster-enhancement (TFCE) with a family-wise error rate (FWE) *p*-value correction to control for multiple comparisons.

## 3. Results

### 3.1. Repetitive Motor Behavior

The outcomes of repetitive motor assessments were previously reported in [24]. These measures are briefly summarized here as these scores were used in fMRI analyses to determine brain regions with functional connectivity differences associated with RRB. In both age cohorts, SH female C58 mice displayed significantly (Bonferroni-corrected, *p* < 0.05) greater repetitive motor behavior than all other groups, with the exception of SH male C58 mice. In both age cohorts, the repetitive behavior of SH male C58 mice was not significantly different from that of EE C58 male mice even though the mean repetitive motor behavior was higher due to individual variation in this group.

### 3.2. Independent Component Analysis

**Figure 1:**
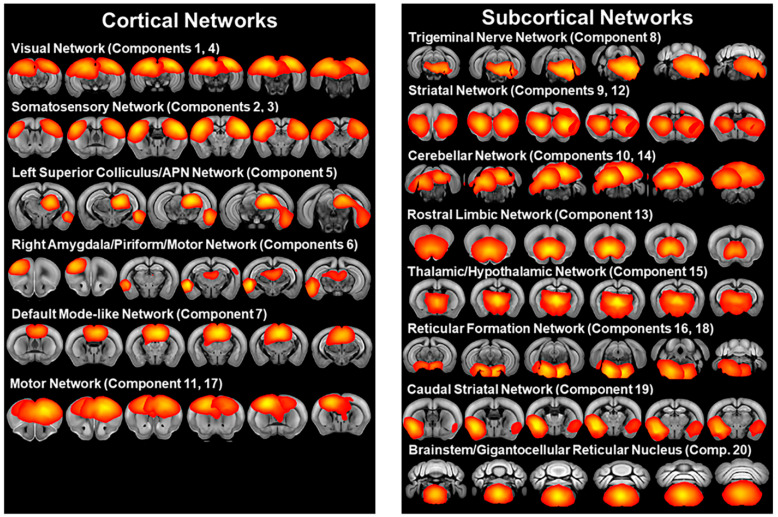
Resting state networks identified by independent component analysis of fMRI scans of C58 and C57 mice at 6 weeks post-weaning including scans from both sexes and housing treatments (n = 78). Networks shown in red to yellow with yellow indicating higher Z scores.

The group ICA of all subjects in the older 6-week post-weaning cohort identified 20 independent components representing resting state networks (Figure 1, Supplementary Table S1), which were broadly consistent with previously published resting state networks [34,35,36,37]. ICA components included bilateral somatosensory, motor, striatal, visual/subiculum, and cerebellar networks. A thalamic/hypothalamic network (component 15) centered on the central medial thalamic nucleus and including the left and right thalamus, hypothalamus, and parts of the midbrain was also identified. ICA components also included a network (component 6) with the highest Z-score in the right lateral amygdala nucleus including regions in the right amygdala, piriform cortex, and primary motor cortex, as well as a network (component 7) with the highest Z-scores in the corpus callosum body and predominantly including the retrosplenial and anterior cingulate cortex resembling the default mode-like network of Sforazzini et al. [36] and Stafford et al. [38]. ICA also detected a network (component 13) with the highest Z-score in the taenia tecta and including the left and right nucleus accumbens, septal nuclei, olfactory areas, and orbital and infralimbic cortices, which we identified as a rostral limbic network similar to Mechling et al. [35]. A caudal striatal network (component 19) centered on the ventral region of the left and right caudal striatum and including the left and right endopiriform nucleus, piriform cortex, basolateral amygdala, and agranular insular area was also identified, which is similar to the salience networks of Sforazzini et al. [36] and Mandino et al. [39]. ICA also identified a network (component 8) in the left brainstem with the highest Z-scores in the principal sensory nucleus of the trigeminal nerve and including nuclei in the reticular formation and parts of the cerebellum. Left and right reticular formation networks (component 16 and 18) centered on the left tegmental reticular nucleus and right pontine reticular nucleus and including reticular formation nuclei were also identified, as well as a brainstem network (component 20) centered on the gigantocellular reticular nucleus and including reticular formation nuclei. ICA components also included a network (component 5) with the highest Z-scores in the left superior colliculus motor regions and anterior pretectal nucleus and including the periaqueductal gray, left posterior complex and lateral posterior thalamic nuclei, left endopiriform nucleus, left entorhinal cortex, and left lateral amygdala nucleus, brain regions which are associated with pain, fear, and defensive behaviors [40,41,42].

**Figure 2:**
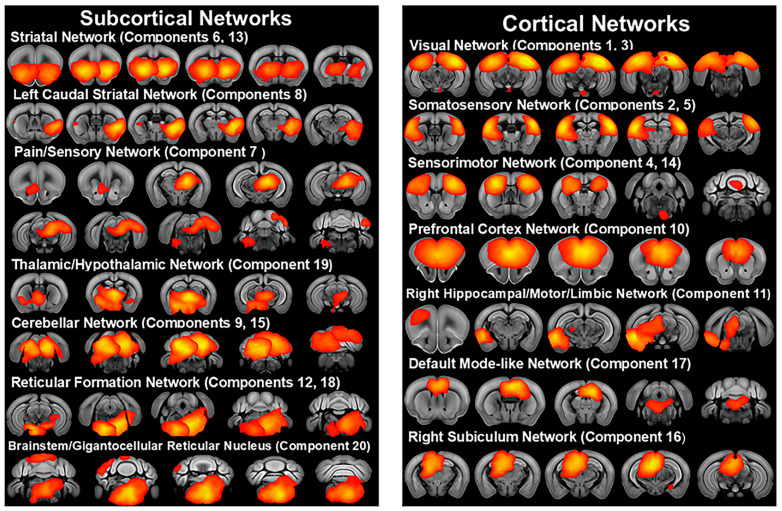
Resting state networks identified by independent component analysis of fMRI scans of C58 and C57 mice at 3 weeks post-weaning including scans from both sexes and housing treatments (n = 18). Networks shown in red to yellow with yellow indicating higher Z scores.

The group ICA of all subjects in the younger 3-week post-weaning cohort identified 20 independent components representing resting state networks (Figure 2, Supplementary Table S2). These networks were generally similar to the older cohort. In contrast to the older cohort, the ICA of the younger cohort included bilateral sensorimotor networks, which combined the motor and somatosensory cortices with the anterior striatum. ICA components also included a left caudal striatal network (component 8) with the highest Z-score in the ventrolateral striatum and covering the ventral portion of the left caudal striatum, as well as including the basolateral amygdala, piriform, endopiriform nucleus, agranular insular cortex, and entorhinal area. This network was similar to the bilateral caudal striatal network of the older cohort. ICA also identified a predominantly subcortical network (component 7), which incorporated pain modulation pathways [41] with sensory and reward pathways, displaying the highest Z-score in the left posterior thalamic complex and including higher order thalamic nuclei and extending into the hippocampus and caudally to the anterior pretectal nucleus, reticular formation nuclei, periaqueductal gray, superior colliculus, cuneiform nucleus, and cerebellar crus I, while also including the nucleus accumbens, primary visual cortex, taenia tecta, anterior olfactory nucleus, and ventral cochlear nucleus. Other networks included a network (component 10), with the highest Z-score in the infralimbic and prelimbic cortices and including the anterior cingulate cortex and orbital cortex, which was similar to the prefrontal network of Sforazzini et al. [36]. A right hippocampal/motor/limbic network (component 11) centered on right CA1 and including the right primary and secondary motor cortex, entorhinal area, caudal ventral striatum, amygdala, subiculum, piriform cortex, agranular insular cortex, periaqueductal gray, superior colliculus deep motor regions, anterior pretectal nucleus, substantia nigra pars reticulata, and midbrain reticular nucleus was also identified. ICA also identified a right subiculum network (component 16) centered on the subiculum and including the retrosplenial area, hippocampus, anterior right thalamus, and superior colliculus.

### 3.3. Mouse Strain Differences in Functional Connectivity

**Figure 3:**
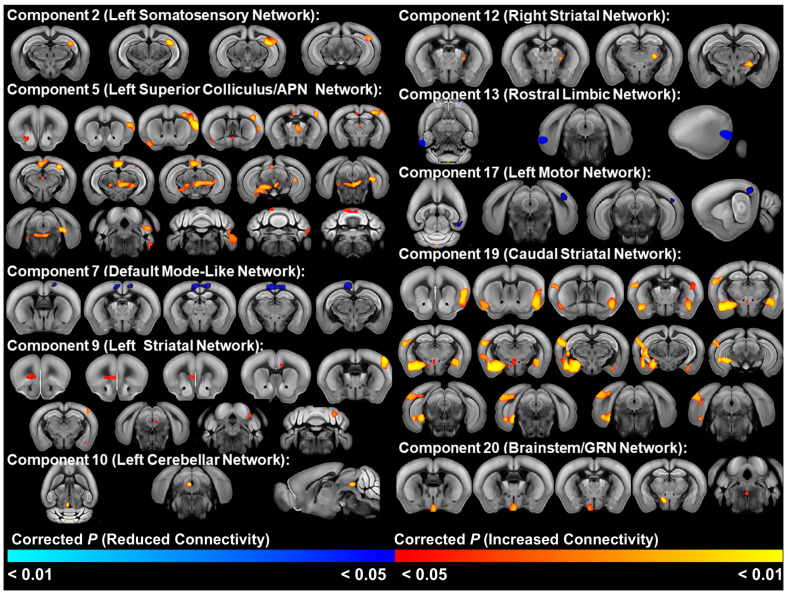
Brain regions with significant differences (FWE-corrected *p* < 0.05) in functional connectivity between C58 and C57 mouse strains in the older 6-week post-weaning cohort. Red and orange areas indicate greater connectivity in C58 mice. Blue indicates decreased connectivity in C58 mice. APN = anterior pretectal nucleus. GRN = gigantocellular reticular nucleus.

The mouse strain *t*-test of ICA components in the older 6-week post-weaning cohort identified significant (FWE-corrected *p* < 0.05) functional connectivity differences in 10 of the 20 ICA components (Figure 3, Supplementary Table S3, Supplementary Figure S1). In 7 of these 10 networks, C58 mice displayed increased functional connectivity, which included the left somatosensory network, a network centered on the left superior colliculus/anterior pretectal nucleus network, left and right striatal networks, caudal striatal network, left cerebellar network, and brainstem/gigantocellular reticular network. Two of these networks, the left superior colliculus/anterior pretectal nucleus network and the caudal striatal network, showed widespread increased functional connectivity with a number of regions throughout the brain in C58 mice, whereas the other five networks exhibited altered connectivity with a smaller number of other brain regions. Reduced functional connectivity was observed in C58 mice in the default mode-like network, rostral limbic network, and the left motor network. The younger cohort did not include multiple mouse strains, precluding a strain comparison for this cohort.

### 3.4. Relationship of RRB to Functional Connectivity

**Figure 4:**
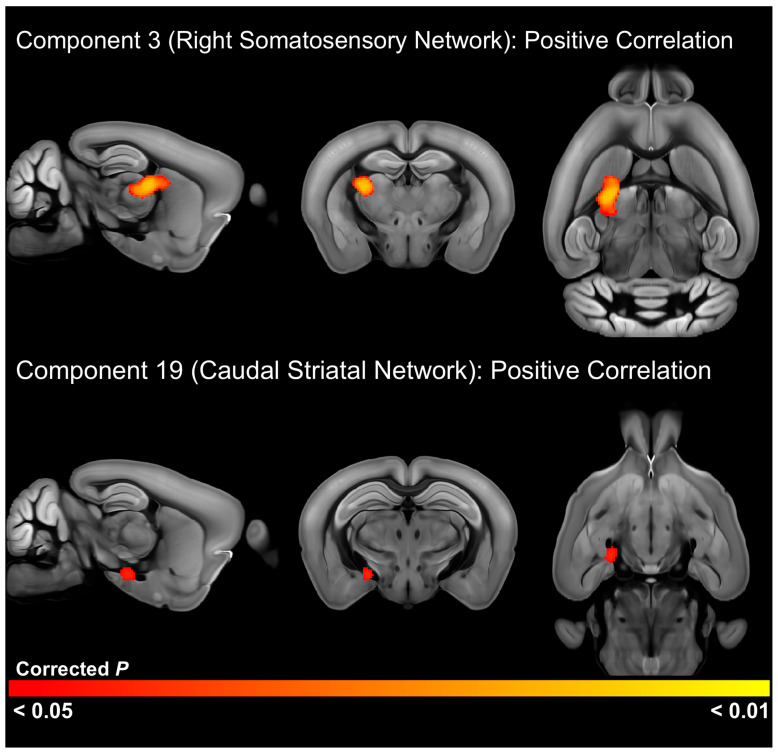
Brain regions with a significant (FWE-corrected *p* < 0.05) positive correlation between functional connectivity and repetitive motor behavior scores in the older 6-week post-weaning cohort.

**Figure 5:**
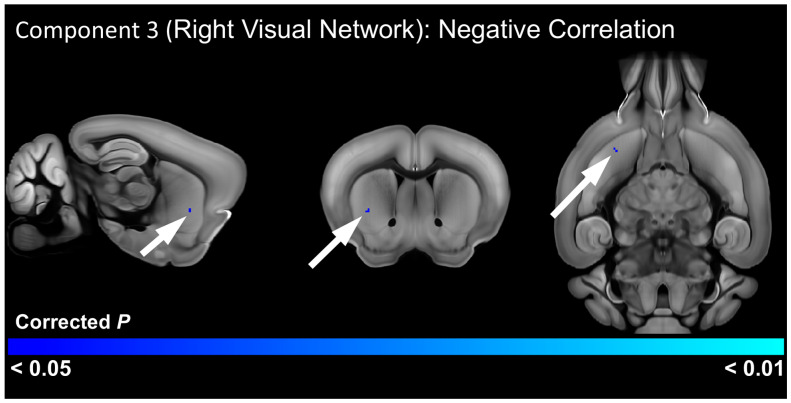
Brain regions with a significant (FWE-corrected *p* < 0.05) negative correlation between functional connectivity and repetitive motor scores in 3-week post-weaning C58 mice. Arrows indicate a small significant region in the right striatum in blue.

To determine resting state networks and brain regions with functional connectivity differences associated with repetitive behavior, we conducted a correlation analysis between repetitive behavior scores and the resting state networks identified by the ICA. In the older cohort, this analysis identified a significant positive correlation (FWE-corrected *p* < 0.05) between the right somatosensory network and a cluster centered on the right reticular nucleus of the thalamus and including other thalamic nuclei, as well as the right dorsomedial striatum (Figure 4, Supplementary Table S4, Supplementary Figure S2). This analysis also detected a significant positive correlation between the caudal striatal network and a cluster centered on white matter tracts in the right hemisphere, including the internal capsule, alveus, and optic tract, but also incorporating the right medial and posterior amygdala nuclei (Figure 4, Supplementary Table S4, Supplementary Figure S2). In the younger cohort, there was a significant negative correlation between RRB scores and functional connectivity of the right visual network with the right ventrolateral striatum (Figure 5, Supplementary Figure S3), suggesting that reduced connectivity between this basal ganglia region and the visual system is associated with RRB.

### 3.5. EE Effects on Functional Connectivity

**Figure 6:**
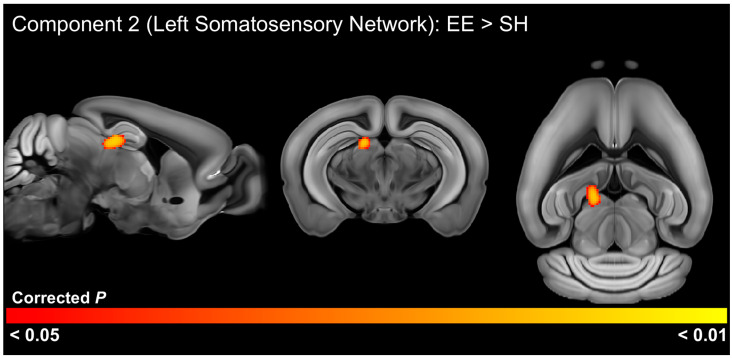
Significant differences (FWE-corrected *p* < 0.05) in functional connectivity between mice housed in environmental enrichment (EE) versus standard housing (SH). Brain regions identified have significantly increased functional connectivity with the left somatosensory network in EE-housed mice in the older 6-week post-weaning cohort.

**Figure 7:**
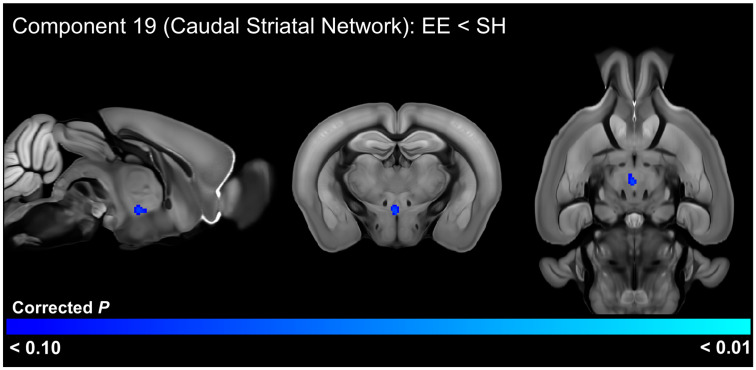
Nearly significant differences (FWE-corrected *p* < 0.10) in functional connectivity between mice housed in environmental enrichment (EE) versus standard housing (SH). Brain regions identified have decreased functional connectivity with the caudal striatal network in EE-housed mice in the older 6-week post-weaning cohort.

In the older cohort, the housing *t*-test of ICA components identified significant (FWE-corrected *p* < 0.05) functional connectivity differences in 1 of the 20 ICA components (Figure 6, Supplementary Table S5, Supplementary Figure S4). This analysis indicated that EE-housed animals had increased connectivity between the left somatosensory network (component 2) with the four pretectal nuclei and regions of the hippocampus. This analysis also detected a trend-level (FWE-corrected *p* < 0.10) decrease in functional connectivity between the caudal striatal network (component 19) with the xiphoid, reuniens, and perireuniens thalamic nuclei, as well as the paraventricular hypothalamic nucleus (Figure 7, Supplementary Table S6, Supplementary Figure S5).

**Figure 8:**
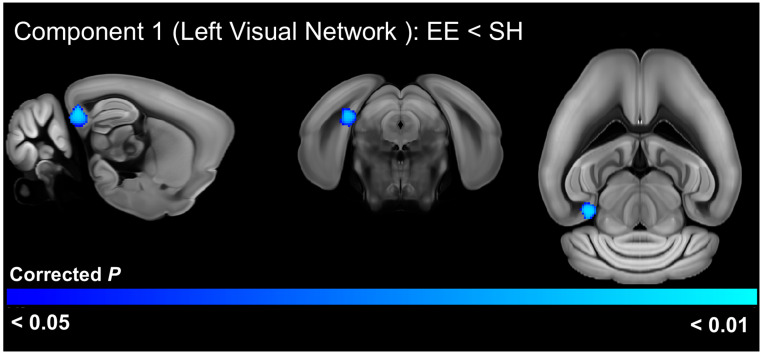
Significant differences (FWE-corrected *p* < 0.05) in functional connectivity between C58 mice at 3 weeks post-weaning housed in environmental enrichment (EE) versus standard housing (SH). Brain regions identified have significantly decreased functional connectivity with the left visual network in EE-housed C58 mice.

A *t*-test comparing the ICA components in mice from the two housing treatments in the younger 3-week post-weaning cohort identified significantly (FWE-corrected *p* < 0.05) reduced connectivity of the left visual network with the right area prostriata and presubiculum, as well as other surrounding brain areas in animals exposed to EE (Figure 8, Supplementary Table S7, Supplementary Figure S6).

## 4. Discussion

This study sought to determine the functional connectivity alterations associated with the increased expression of RRB in C58 mice, as well as its attenuation by EE. Understanding the neurocircuitry alterations responsible for the robust RRB phenotype in this inbred mouse strain and the neural alterations underlying how EE reduces RRB may offer new insights into the neurobiology and treatment of RRB in autism and other disorders. Our results revealed widespread functional connectivity differences between the C58 and C57 mouse strains. Half of the resting state networks identified with ICA in the older, 6-week post-weaning cohort displayed significant strain differences. Furthermore, RRB was associated with altered connectivity of somatosensory, visual, and basal ganglia networks and their connections to the reticular thalamic nucleus and lateral thalamic nuclei, striatum, and the medial and posterior amygdala nuclei. Consistent with past studies, EE reduced strain differences in repetitive motor behavior, particularly in female C58 mice [15,16]. These EE-induced RRB reductions were accompanied by reduced connectivity between the visual network with hippocampal regions and area prostriata in the younger cohort, as well as increased connectivity of somatosensory pathways with pretectal nuclei and hippocampal regions in the older cohort.

In general, strain differences predominantly reflected hyperconnectivity of functional networks in the C58 mouse strain, although the default mode-like, left motor, and rostral limbic networks displayed hypoconnectivity. Two networks stood out for their widespread hyperconnectivity with other brain regions: a network in the caudal striatum and a network centered on the superior colliculus and anterior pretectal nucleus. The caudal striatum, which is innervated by limbic and sensory processing regions, plays a key role in sensory integration by associating sensory input with salience information [43,44,45] and has been theorized to mediate the reinforcement learning of avoidance of threatening stimuli [43,46]. Interestingly, Menegas et al. [46] demonstrated that dopamine neurons in the caudal striatum responded only to novel or high-intensity sensory stimuli and induced avoidance, leading them to speculate that these neurons encoded external threat. Furthermore, they showed that ablation of dopamine neurons in this region led to reduced avoidance of aversive and novel stimuli over time, suggesting dopamine neurons in this region are necessary for maintenance of avoidance responses. These findings were extended by Krüttner et al. [47], who demonstrated that shank 3 mice, an autism animal model, exhibited context-dependent avoidance of objects and conspecifics, which was mediated by increased dopamine release in the caudal striatum. Similarly, Akiti et al. [43] observed dopamine was released in the caudal striatum at the beginning of retreat during exploratory behavior and that mouse avoidance behavior of novel objects was strongly correlated with individual variation in dopamine levels in this region. This led them to propose a reinforcement learning model in which dopamine in the caudal striatum signals threat prediction during exploration of novel stimuli, eventually determining habituation or avoidance. Thus, the hyperconnectivity of a caudal striatal network with sensory processing and amygdala regions in C58 mice may suggest altered salience and threat perception. Increased connectivity of this network with sensory regions in C58 mice could also reflect altered sensory perception in which sensory stimuli are more frequently perceived as highly intense or novel. Furthermore, hyperconnectivity of this network with hippocampal areas and the thalamic nucleus reuniens, which is important in encoding contextual memories [48], suggests altered sensory learning and memory in C58 mice.

C58 mice also exhibited extensive hyperconnectivity in a network centered on the superior colliculus and anterior pretectal nucleus. Hyperconnected regions included hippocampal regions, the somatosensory cortex, anterior cingulate and retrosplenial cortices, zona incerta, periaqueductal gray, nucleus accumbens, and substantia nigra, as well as various medial, midline, posterior, and intralaminar thalamic nuclei. Motor regions including the motor cortex, reticular nuclei including the pedunculopontine nucleus, and motor regions of the cerebellum were also hyperconnected to this network. Interestingly, many of these regions are associated with pain perception [41,49,50]. Painful stimuli in humans and animals are commonly associated with activation in the periaqueductal gray, anterior pretectal nucleus, medial thalamic nuclei, sensory and motor cortices, restrosplenial cortex, anterior cingulate cortex, and motor cerebellar regions [51,52,53]. In addition, electrical stimulation of the anterior pretectal nucleus, periaqueductal gray, and retrosplenial cortex have been shown to inhibit responses to painful stimuli [50,54,55]. Antinociceptive pathways from the anterior pretectal nucleus are the result of both descending pathways to the spinal cord via the lateral paragigantocellular nucleus and pedunculopontine nucleus/deep mesencephalic nucleus, as well as inhibition of thalamic nuclei both directly and indirectly through connections with the zona incerta [41,56]. Thus, the hyperconnectivity we observed in this network may reflect altered pain processing in C58 mice. This conclusion is further supported by other resting state networks that exhibited altered connectivity with pain-associated regions in C58 mice. These included increased connectivity between the cerebellar network and the periaqueductal gray, as well as increased connectivity between the right striatal network with the zona incerta and the left reticular and ventral posterolateral and posteromedial thalamic nuclei. The ventral posterolateral and posteromedial nuclei are lateral thalamic nuclei that convey somatosensory information, including pain, from the body via the spinothalamic tract and the face via the trigeminothalamic tract, to the somatosensory cortex [57,58,59]. The reticular thalamic nucleus regulates thalamic activity and the passage of sensory information, including pain, between the thalamus and cortex, and has been shown to induce antinociception via connections to the ventral posterolateral and posteromedial nuclei [57,60,61].

Alternatively, the connectivity differences C58 mice displayed in the superior colliculus/anterior pretectal nucleus network may reflect altered avoidance/defensive behaviors. The superior colliculus is a key region for sensorimotor integration and attention with roles in multiple behavioral circuits including avoidance, defensive, and appetitive behaviors [62,63,64]. The superior colliculus, periaqueductal gray, and pedunculopontine nucleus are part of an avoidance circuit, which is modulated by the substantia nigra pars reticulata [65,66,67].

Despite widespread strain differences, RRB was correlated with alterations in a small number of networks. In the older cohort, RRB was positively correlated with functional connectivity of the right somatosensory network with a cluster centered on the right reticular thalamic nucleus and including posterior and lateral thalamic nuclei such as the ventral posterolateral and posteromedial nuclei. As discussed previously, these thalamic nuclei are associated with relaying somatosensory information to the cortex and play important roles in pain perception. In the older cohort, RRB was also positively correlated with connectivity between a network in the caudal striatum with the medial and posterior amygdala nuclei. These nuclei represent a subset of the regions hyperconnected with this network in C58 mice in the strain comparison. These amygdala nuclei convey olfactory/pheromone information to the hypothalamus in association with mediating reproductive and aggressive social behaviors [68,69]. Interestingly, activation of glutamatergic medial amygdala neurons can both inhibit social behavior and induce stereotypic behaviors [70]. This suggests a role for altered amygdala function in driving RRB, perhaps in association with aberrant olfactory processing or salience and threat perception due to altered connectivity with the caudal striatum.

In the younger cohort, RRB was negatively associated with connectivity between the right visual network and a small region in the right ventrolateral striatum. The ventrolateral striatum is involved in goal-directed movements and action initiation [71]. It has previously been associated with stereotypic behavior [72,73,74]. This brain region has also been demonstrated to play a role in nociception [75], addiction [76], and reward-based decision making [77]. Interestingly, it has also been associated with dopamine release in animals housed in impoverished conditions that actively seek aversive stimuli as a type of self-stimulation [78]. Thus, RRB appears to be associated with altered sensory function across multiple sensory modalities, including in pain or somatosensory processing regions, visual pathways, and regions important in the interpretation of olfactory social signals.

Previous studies have noted strong associations between RRB and sensory processing alterations in both animal models of RRB and ASD, as well as humans with ASD [79,80,81,82], suggesting a related pathophysiology between sensory processing deficits and RRB. For example, sensory deficits, including altered sensitivity to painful stimuli, are commonly observed in animal models of ASD that also exhibit RRB [83]. In addition, clinical measures of RRB and sensory processing deficits are often highly correlated in human autism studies [80,82,84,85]. Interestingly, Schulz and Stevenson [80] observed that sensory hypersensitivity was correlated with increased RRB in both ASD and TD children. They further observed that this correlation held across all sensory modalities and all repetitive behavior subtypes. Further support for a link between sensory hypersensitivity and RRB comes from interviews of autistic individuals. Manor-Binyamini and Schreiber-Divon [86] interviewed verbal adults with ASD and found that many participants engaged in RRBs to cope with hypersensitivity to sensory stimuli, which were often perceived as painful or threatening. While studies providing mechanistic links between RRB and sensory deficits are sparse, studies in the BTBR mouse model of ASD suggest that alterations in the nicotinic cholinergic system may play a role in both altered pain sensitivity and RRB. Nicotine has been demonstrated to both reduce RRB and alter pain sensitivity in BTBR mice, which have reduced expression of some nicotinic acetylcholine receptor subunits [81,87]. Gogolla et al. [79] linked multisensory integration deficits in BTBR mice to reduced parvalbumin inhibitory neurons and altered perineuronal nets in the insular cortex. They further found that diazepam administration in juvenile mice rescued inhibitory circuitry while improving both multisensory integration and reducing RRB, suggesting that both sensory deficits and RRB result from a cortical excitation/inhibition balance in BTBR mice. Additional evidence for a shared underlying mechanism comes from a human DTI study. Wolff et al. [82] determined that high fractional anisotropy in 6-month-old infants in the genu of the corpus callosum were predictive of both sensory deficits and increased repetitive behaviors at the age of 2 years in children later diagnosed with ASD. Our work further adds to this evidence of a connection between RRB and sensory deficits by suggesting that RRB in C58 mice is associated with altered functional connectivity in sensory networks, perhaps due to sensations of pain or threat caused by deficits in sensory integration or altered sensory thresholds as reported in human subjects. Future work should investigate whether C58 mice exhibit altered inhibitory and nicotinic cholinergic circuitry similar to BTBR mice and the relationship between sensory stimuli and RRB.

Our findings are also consistent with other studies linking RRB to basal ganglia alterations. Basal ganglia regions, including the striatum, have been commonly implicated in RRB in both C58 mice and other animal models [4,88]. Past studies have shown strong associations between RRB in C58 mice and alterations in the indirect basal ganglia pathway [15,89,90]. The indirect pathway is believed to play an inhibitory role in behavior through thalamic inhibition [91,92]. For instance, in one of the few neuroimaging studies of RRB in C58 mice, Wilkes et al. [90] observed a correlation between RRB and the volume of the subthalamic nucleus and striatum, as well as the crus II of the cerebellum. Studies have also shown that direct manipulation of the indirect basal ganglia pathway can reduce RRB in C58 mice [89] and other animal models [21,93]. Interestingly, our findings suggest the importance of connectivity between sensory networks and the striatum in RRB rather than between the striatum and motor regions, suggesting RRB may result from aberrant integration of sensory information into basal ganglia pathways.

Despite the widespread neural changes reported for EE in the broader literature, EE-housed mice in this study exhibited changes in relatively few resting state networks. In older mice, EE induced hyperconnectivity of the left somatosensory network with right hippocampal and pretectal nuclei, particularly the anterior pretectal nucleus. Somatosensory inputs to the hippocampus aid in the formation of episodic memories and spatial learning/memory [94]. The anterior pretectal nucleus plays an important role in analgesia and behavioral responses to pain [41,50]. Thus, these differences may reflect a strengthening of an antinociceptive pathway or alteration of how somatosensory and pain information is integrated during learning and memory. EE-housed mice also displayed a trend towards hypoconnectivity of the caudal striatum network with the xiphoid, reuniens, and perireuniens thalamic nuclei, as well as the paraventricular hypothalamic nucleus. Given the role of these thalamic nuclei in freezing and aggressive behaviors in response to visual threats and the role of the paraventricular thalamus in stress response, it is possible that these EE-induced changes in the caudal striatum network reflect changes in threat perception and response [95,96].

In the younger cohort, EE resulted in reduced visual network connectivity. EE reduced the connectivity of the left visual network with the right presubiculum and right area prostriata, a region that facilitates behavioral responses to quickly moving and therefore threatening visual stimuli [97,98]. These results suggest alterations in visual processing and spatial learning may be related to EE’s attenuation of RRB. Alternatively, they may reflect an EE reversal of a visual deficit or a lack of peripheral visual stimulation due to reduced RRB under EE conditions.

Previous investigations of EE in other animal models have shown it can induce a wide range of neural alterations including neurogenesis, increased neuron survival, gliogenesis, increases in dendritic arborization and synaptic plasticity, increased neurotrophins, reduced neuroinflammation, increased myelination, and altered epigenetic and genetic expression [9,99,100]. Of the few studies examining the neural mechanisms underlying the beneficial effects of EE on repetitive motor behavior, most have linked RRB decreases to increases in neuronal activation and synaptic plasticity within the indirect basal ganglia pathway [6]. While our findings implicating sensory networks may at first seem surprising in this context, it should be noted that these previous studies of EE effects on RRB focused only on a few brain regions, predominantly within the basal ganglia. In contrast, our investigation took a more agnostic data-driven and brain-wide approach. Moving beyond the RRB literature, studies have demonstrated that EE can reduce sensory deficits [101,102,103] and reduce pain sensitivity [104,105,106]. Thus, EE may reduce RRB indirectly through a reduction in sensory hypersensitivity or increase in pain tolerance. Our findings associating RRB scores and EE with connectivity between the somatosensory network and thalamic and pretectal nuclei involved in pain pathways suggest this, as does the involvement of the same sensory networks in both RRB and EE. Alternatively, EE has also been shown to rescue cortical excitation/inhibition imbalance [103,107], which has been proposed as a common mechanism underlying sensory dysfunction and RRB in BTBR mice [79]. Thus, our EE results could reflect EE-induced sensory alterations that happen to coincide with RRB reduction via a common mechanism. Of further note, our recent DTI study of EE effects in C58 mice found that EE induced widespread alterations in gray matter microstructure throughout the brain, including in sensory regions displaying a negative association between fractional anisotropy and RRB [24]. Taken together, these neuroimaging studies suggest an important role for functional and structural alterations in sensory regions in RRB development and its attenuation by EE in C58 mice, particularly in the visual and somatosensory cortices.

Several important study limitations should be noted. The lack of a control strain in the younger cohort prevented us from examining strain differences in this age cohort. Additionally, MRI scans of younger mice may have provided additional information about early RRB development, although scanning even younger mice may have been prohibitive due to their small size. It is possible that there are earlier strain and housing differences in functional connectivity, which may not be evident at later timepoints, that are relevant during the development of RRB. Future longitudinal and crossover studies would be beneficial to allow the measurement of individual changes over time and observe changes in brain-behavior relationships before and after housing treatments. Though both sexes were included in this study, sex differences were not specifically examined. Thus, sex-specific associations with RRB and sex-specific strain and EE connectivity alterations were not identified. This study also did not examine brain tissues to identify the neural alterations underlying the functional connectivity differences observed. It should also be noted that C58 mice are considered models of RRB rather than an ASD model. Future studies should investigate if these results can be replicated in other animal models of RRB and ASD.

## 5. Conclusions

Taken together, our results suggest RRB in C58 mice results from sensory processing alterations across multiple sensory domains. Extensive strain differences in functional network connectivity, particularly of sensory processing and basal ganglia networks, may support the development of RRB in C58 mice, although there was little overlap between strain differences and the connectivity differences correlated with RRB. The one exception to this was the caudal striatal network, which displayed alterations associated with both strain differences and RRB measures. EE may attenuate RRB in C58 mice by increasing connectivity between antinociceptive regions and the somatosensory cortex. EE may also be related to reduced connectivity between visual pathways and a region involved in reflexive responses to fast-moving visual stimuli. Our findings add to a growing body of evidence suggesting a connection between RRB development, sensory processing deficits, and pain. Future studies should investigate the relationship between sensory processing and RRB development, as well as the relationship between pain circuitry and RRB. This study identifies multiple brain regions and networks with altered functional connectivity in relation to strain, EE, and RRB associations that may serve as useful targets for future pharmacological and behavioral interventions.

## Figures and Tables

**Table 1:** Demographics of mice included in behavioral assessments (and fMRI scans) for each age cohort.

Age Group	Housing Type	C58 Females	C58 Males	C57 Females	C57 Males
6 weeks post-weaning	EE	8 (8)	12 (10)	12 (12)	9 (9)
	SH	11 (11)	12 (10)	11 (11)	7 (7)
3 weeks post-weaning	EE	6 (6)	4 (4)	Not included	Not included
	SH	6 (5)	6 (3)	Not included	Not included

## Data Availability

All raw MRI images and associated behavioral data are openly available on OpenNeuro (https://openneuro.org/; accession numbers ds005635 and ds005636).

## Supplementary Materials

The following supporting information can be downloaded at: https://www.mdpi.com/article/10.3390/cells13231933/s1, Figure S1: Mean Z values for significant network differences between mouse strains. Figure S2: Correlation between subject Z values and repetitive behavior (RRB) scores for functional networks with significant RRB associations in the older 6-week post-weaning cohort. Figure S3: Correlation between subject Z values and repetitive behavior (RRB) scores for functional networks with significant RRB associations in the younger 3-week post-weaning cohort. Figure S4: Mean Z values for significant network differences between housing conditions in the older 6-week post-weaning cohort. Figure S5: Mean Z values for nearly significant network differences between housing conditions in the older 6-week post-weaning cohort. Figure S6: Mean Z values for significant network differences between housing conditions in the younger 3-week post-weaning cohort. Table S1: Resting state networks resulting from an independent component analysis of the older mouse cohort. Table S2: Resting state networks resulting from an independent component analysis of the younger mouse cohort. Table S3: Resting state networks and brain regions with significant functional connectivity differences between mouse strains in the older 6-week post-weaning cohort. Table S4: Resting state networks with significant functional connectivity differences correlated with repetitive motor scores in C58 and C57 mice at 6 weeks post-weaning. Table S5: Resting state networks and brain regions with significant functional connectivity differences between housing conditions in the older 6-week post-weaning cohort. Table S6: Resting state networks and brain regions with nearly significant functional connectivity differences between housing conditions in the older 6-week post-weaning cohort. Table S7: Resting state networks and brain regions with significant functional connectivity differences between housing conditions in C58 mice at 3 weeks post-weaning.

## References

[1] Bodfish J.W., Symons F.J., Parker D.E., Lewis M.H. (2000). Varieties of repetitive behavior in autism: Comparisons to mental retardation. J. Autism Dev. Disord..

[2] Turner M. (1999). Annotation: Repetitive behaviour in autism: A review of psychological research. J. Child Psychol. Psychiatry.

[3] Lewis M., Kim S.J. (2009). The pathophysiology of restricted repetitive behavior. J. Neurodev. Disord..

[4] Wilkes B.J., Lewis M.H. (2018). The neural circuitry of restricted repetitive behavior: Magnetic resonance imaging in neurodevelopmental disorders and animal models. Neurosci. Biobehav. Rev..

[5] Tian J., Gao X., Yang L. (2022). Repetitive restricted behaviors in autism spectrum disorder: From mechanism to development of therapeutics. Front. Neurosci..

[6] Farmer A.L., Lewis M.H. (2023). Reduction of restricted repetitive behavior by environmental enrichment: Potential neurobiological mechanisms. Neurosci. Biobehav. Rev..

[7] Forbes T.A., Gallo V. (2017). All wrapped up: Environmental effects on myelination. Trends Neurosci..

[8] Hannan A.J. (2014). Environmental enrichment and brain repair: Harnessing the therapeutic effects of cognitive stimulation and physical activity to enhance experience-dependent plasticity. Neuropathol. Appl. Neurobiol..

[9] Hirase H., Shinohara Y. (2014). Transformation of cortical and hippocampal neural circuit by environmental enrichment. Neuroscience.

[10] Canario E., Chen D., Biswal B. (2021). A review of resting-state fMRI and its use to examine psychiatric disorders. Psychoradiology.

[11] Delmonte S., Gallagher L., O’hanlon E., McGrath J., Balsters J.H. (2013). Functional and structural connectivity of frontostriatal circuitry in Autism Spectrum Disorder. Front. Hum. Neurosci..

[12] Uddin L.Q., Supekar K., Lynch C.J., Khouzam A., Phillips J., Feinstein C., Ryali S., Menon V. (2013). Salience network–based classification and prediction of symptom severity in children with autism. JAMA Psychiatry.

[13] Weng S.J., Wiggins J.L., Peltier S.J., Carrasco M., Risi S., Lord C., Monk C.S. (2010). Alterations of resting state functional connectivity in the default network in adolescents with autism spectrum disorders. Brain Res..

[14] Sforazzini F., Bertero A., Dodero L., David G., Galbusera A., Scattoni M.L., Pasqualetti M., Gozzi A. (2016). Altered functional connectivity networks in acallosal and socially impaired BTBR mice. Brain Struct. Funct..

[15] Lewis M.H., Lindenmaier Z., Boswell K., Edington G., King M.A., Muehlmann A.M. (2018). Subthalamic nucleus pathology contributes to repetitive behavior expression and is reversed by environmental enrichment. Genes Brain Behav..

[16] Muehlmann A.M., Edington G., Mihalik A.C., Buchwald Z., Koppuzha D., Korah M., Lewis M.H. (2012). Further characterization of repetitive behavior in C58 mice: Developmental trajectory and effects of environmental enrichment. Behav. Brain Res..

[17] Curry-Pochy L.S., Kravetz Z., Feinstein J., Yaffe B., Tanios V., Makar J., Lewis M.H. (2020). Differential consequences of habitual responding in a mouse model of repetitive behavior. Behav. Neurosci..

[18] Whitehouse C.M., Curry-Pochy L.S., Shafer R., Rudy J., Lewis M.H. (2017). Reversal learning in C58 mice: Modeling higher order repetitive behavior. Behav. Brain Res..

[19] Bechard A.R., Bliznyuk N., Lewis M.H. (2017). The development of repetitive motor behaviors in deer mice: Effects of environmental enrichment, repeated testing, and differential mediation by indirect basal ganglia pathway activation. Dev. Psychobiol..

[20] Bechard A.R., Cacodcar N., King M.A., Lewis M.H. (2016). How does environmental enrichment reduce repetitive motor behaviors? Neuronal activation and dendritic morphology in the indirect basal ganglia pathway of a mouse model. Behav. Brain Res..

[21] Tanimura Y., Vaziri S., Lewis M.H. (2010). Indirect basal ganglia pathway mediation of repetitive behavior: Attenuation by adenosine receptor agonists. Behav. Brain Res..

[22] Turner C.A., Lewis M.H., King M.A. (2003). Environmental enrichment: Effects on stereotyped behavior and dendritic morphology. Dev. Psychobiol..

[23] Turner C.A., Yang M.C., Lewis M.H. (2002). Environmental enrichment: Effects on stereotyped behavior and regional neuronal metabolic activity. Brain Res..

[24] Farmer A.L., Febo M., Wilkes B.J., Lewis M.H. (2024). Environmental enrichment reduces restricted repetitive behavior by altering gray matter microstructure. PLoS ONE.

[25] Yang Z., Zhu T., Pompilus M., Fu Y., Zhu J., Arjona K., Arja R.D., Grudny M.M., Plant H.D., Bose P. (2021). Compensatory functional connectome changes in a rat model of traumatic brain injury. Brain Commun..

[26] Cox R.W. (1996). AFNI: Software for analysis and visualization of functional magnetic resonance neuroimages. Comput. Biomed. Res..

[27] Jenkinson M., Bannister P., Brady M., Smith S. (2002). Improved optimization for the robust and accurate linear registration and motion correction of brain images. Neuroimage.

[28] Klein A., Andersson J., Ardekani B.A., Ashburner J., Avants B., Chiang M.C., Christensen G.E., Collins L., Gee J., Hellier P. (2009). Evaluation of 14 nonlinear deformation algorithms applied to human brain MRI registration. Neuroimage.

[29] Chou N., Wu J., Bingren J.B., Qiu A., Chuang K.H. (2011). Robust automatic rodent brain extraction using 3-D pulse-coupled neural networks (PCNN). IEEE Trans. Image Process..

[30] Yushkevich P.A., Piven J., Hazlett H.C., Smith R.G., Ho S., Gee J.C., Gerig G. (2006). User-guided 3D active contour segmentation of anatomical structures: Significantly improved efficiency and reliability. Neuroimage.

[31] Lein E.S., Hawrylycz M.J., Ao N., Ayres M., Bensinger A., Bernard A., Boe A.F., Boguski M.S., Brockway K.S., Byrnes E.J. (2007). Genome-wide atlas of gene expression in the adult mouse brain. Nature.

[32] Beckmann C.F., DeLuca M., Devlin J.T., Smith S.M. (2005). Investigations into resting-state connectivity using independent component analysis. Philos. Trans. R. Soc. B Biol. Sci..

[33] Beckmann C.F., Mackay C.E., Filippini N., Smith S.M. (2009). Group comparison of resting-state FMRI data using multi-subject ICA and dual regression. Neuroimage.

[34] Jonckers E., Van Audekerke J., De Visscher G., Van der Linden A., Verhoye M. (2011). Functional connectivity fMRI of the rodent brain: Comparison of functional connectivity networks in rat and mouse. PLoS ONE.

[35] Mechling A.E., Hübner N.S., Lee H.L., Hennig J., von Elverfeldt D., Harsan L.A. (2014). Fine-grained mapping of mouse brain functional connectivity with resting-state fMRI. Neuroimage.

[36] Sforazzini F., Schwarz A.J., Galbusera A., Bifone A., Gozzi A. (2014). Distributed BOLD and CBV-weighted resting-state networks in the mouse brain. Neuroimage.

[37] Zerbi V., Grandjean J., Rudin M., Wenderoth N. (2015). Mapping the mouse brain with rs-fMRI: An optimized pipeline for functional network identification. Neuroimage.

[38] Stafford J.M., Jarrett B.R., Miranda-Dominguez O., Mills B.D., Cain N., Mihalas S., Lahvis G.P., Lattal K.M., Mitchell S.H., David S.V. (2014). Large-scale topology and the default mode network in the mouse connectome. Proc. Natl. Acad. Sci. USA.

[39] Mandino F., Vrooman R.M., Foo H.E., Yeow L.Y., Bolton T.A., Salvan P., Teoh C.L., Lee C.Y., Beauchamp A., Luo S. (2022). A triple-network organization for the mouse brain. Mol. Psychiatry.

[40] Fanselow M.S. (1994). Neural organization of the defensive behavior system responsible for fear. Psychon. Bull. Rev..

[41] Genaro K., Prado W.A. (2021). The role of the anterior pretectal nucleus in pain modulation: A comprehensive review. Eur. J. Neurosci..

[42] Wei P., Liu N., Zhang Z., Liu X., Tang Y., He X., Wu B., Zhou Z., Liu Y., Li J. (2015). Processing of visually evoked innate fear by a non-canonical thalamic pathway. Nat. Commun..

[43] Akiti K., Tsutsui-Kimura I., Xie Y., Mathis A., Markowitz J.E., Anyoha R., Datta S.R., Mathis M.W., Uchida N., Watabe-Uchida M. (2022). Striatal dopamine explains novelty-induced behavioral dynamics and individual variability in threat prediction. Neuron.

[44] Hunnicutt B.J., Jongbloets B.C., Birdsong W.T., Gertz K.J., Zhong H., Mao T. (2016). A comprehensive excitatory input map of the striatum reveals novel functional organization. eLife.

[45] Valjent E., Gangarossa G. (2021). The tail of the striatum: From anatomy to connectivity and function. Trends Neurosci..

[46] Menegas W., Akiti K., Amo R., Uchida N., Watabe-Uchida M. (2018). Dopamine neurons projecting to the posterior striatum reinforce avoidance of threatening stimuli. Nat. Neurosci..

[47] Krüttner S., Falasconi A., Valbuena S., Galimberti I., Bouwmeester T., Arber S., Caroni P. (2022). Absence of familiarity triggers hallmarks of autism in mouse model through aberrant tail-of-striatum and prelimbic cortex signaling. Neuron.

[48] Ramanathan K.R., Jin J., Giustino T.F., Payne M.R., Maren S. (2018). Prefrontal projections to the thalamic nucleus reuniens mediate fear extinction. Nat. Commun..

[49] Guo F., Du Y., Qu F.H., Lin S.D., Chen Z., Zhang S.H. (2022). Dissecting the neural circuitry for pain modulation and chronic pain: Insights from optogenetics. Neurosci. Bull..

[50] Rees H., Roberts M.H.T. (1993). The anterior pretectal nucleus: A proposed role in sensory processing. Pain.

[51] Hess A., Sergejeva M., Budinsky L., Zeilhofer H.U., Brune K. (2007). Imaging of hyperalgesia in rats by functional MRI. Eur. J. Pain.

[52] Hudson A.J. (2000). Pain perception and response: Central nervous system mechanisms. Can. J. Neurol. Sci..

[53] Peyron R., Laurent B., Garcia-Larrea L. (2000). Functional imaging of brain responses to pain. A review and meta-analysis. Clin. Neurophysiol..

[54] Genaro K., Prado W.A. (2016). Neural correlates of the antinociceptive effects of stimulating the anterior pretectal nucleus in rats. J. Pain.

[55] Rossaneis A.C., Reis G.M., Prado W.A. (2011). Stimulation of the occipital or retrosplenial cortex reduces incision pain in rats. Pharmacol. Biochem. Behav..

[56] Giber K., Slézia A., Bokor H., Bodor Á.L., Ludányi A., Katona I., Acsády L. (2008). Heterogeneous output pathways link the anterior pretectal nucleus with the zona incerta and the thalamus in rat. J. Comp. Neurol..

[57] Groh A., Krieger P., Mease R.A., Henderson L. (2018). Acute and chronic pain processing in the thalamocortical system of humans and animal models. Neuroscience.

[58] Potes C.S., Neto F.L., Castro-Lopes J.M. (2006). Inhibition of pain behavior by GABAB receptors in the thalamic ventrobasal complex: Effect on normal rats subjected to the formalin test of nociception. Brain Res..

[59] Wang L.H., Ding W.Q., Sun Y.G. (2022). Spinal ascending pathways for somatosensory information processing. Trends Neurosci..

[60] Liu P.F., Wang Y., Xu L., Xiang A.F., Liu M.Z., Zhu Y.B., Jia X., Zhang R., Li J.-B., Zhang L. (2022). Modulation of itch and pain signals processing in ventrobasal thalamus by thalamic reticular nucleus. Iscience.

[61] Zhang C., Chen R.X., Zhang Y., Wang J., Liu F.Y., Cai J., Liao F., Xu F., Yi M., Wan Y. (2017). Reduced GABAergic transmission in the ventrobasal thalamus contributes to thermal hyperalgesia in chronic inflammatory pain. Sci. Rep..

[62] Isa K., Sooksawate T., Kobayashi K., Kobayashi K., Redgrave P., Isa T. (2020). Dissecting the tectal output channels for orienting and defense responses. ENeuro.

[63] Ito S., Feldheim D.A. (2018). The mouse superior colliculus: An emerging model for studying circuit formation and function. Front. Neural Circuits.

[64] Wheatcroft T., Saleem A.B., Solomon S.G. (2022). Functional organisation of the mouse superior colliculus. Front. Neural Circuits.

[65] Almada R.C., Genewsky A.J., Heinz D.E., Kaplick P.M., Coimbra N.C., Wotjak C.T. (2018). Stimulation of the nigrotectal pathway at the level of the superior colliculus reduces threat recognition and causes a shift from avoidance to approach behavior. Front. Neural Circuits.

[66] Evans D.A., Stempel A.V., Vale R., Ruehle S., Lefler Y., Branco T. (2018). A synaptic threshold mechanism for computing escape decisions. Nature.

[67] Hormigo S., Vega-Flores G., Rovira V., Castro-Alamancos M.A. (2019). Circuits that mediate expression of signaled active avoidance converge in the pedunculopontine tegmentum. J. Neurosci..

[68] Raam T., Hong W. (2021). Organization of neural circuits underlying social behavior: A consideration of the medial amygdala. Curr. Opin. Neurol..

[69] Yamaguchi T., Wei D., Song S.C., Lim B., Tritsch N.X., Lin D. (2020). Posterior amygdala regulates sexual and aggressive behaviors in male mice. Nat. Neurosci..

[70] Hong W., Kim D.W., Anderson D.J. (2014). Antagonistic control of social versus repetitive self-grooming behaviors by separable amygdala neuronal subsets. Cell.

[71] Tsutsui-Kimura I., Natsubori A., Mori M., Kobayashi K., Drew M.R., de Kerchove d’Exaerde A., Mimura M., Tanaka K.F. (2017). Distinct roles of ventromedial versus ventrolateral striatal medium spiny neurons in reward-oriented behavior. Curr. Biol..

[72] Chartoff E.H., Marck B.T., Matsumoto A.M., Dorsa D.M., Palmiter R.D. (2001). Induction of stereotypy in dopamine-deficient mice requires striatal D1 receptor activation. Proc. Natl. Acad. Sci. USA.

[73] Delfs J.M., Kelley A.E. (1990). The role of D1 and D2 dopamine receptors in oral stereotypy induced by dopaminergic stimulation of the ventrolateral striatum. Neuroscience.

[74] Yeghiayan S.K., Kelley A.E. (1995). Serotonergic stimulation of the ventrolateral striatum induces orofacial stereotypy. Pharmacol. Biochem. Behav..

[75] Natsubori A., Miyazawa M., Kojima T., Honda M. (2023). Region-specific involvement of ventral striatal dopamine D2 receptor-expressing medium spiny neurons in nociception. Neurosci. Res..

[76] Mukherjee D., Gonzales B.J., Ashwal-Fluss R., Turm H., Groysman M., Citri A. (2021). Egr2 induction in spiny projection neurons of the ventrolateral striatum contributes to cocaine place preference in mice. eLife.

[77] Thapa R., Gruber A.J. (2018). Lesions of ventrolateral striatum eliminate lose-shift but not win-stay behaviour in rats. Neurobiol. Learn. Mem..

[78] Yawata Y., Shikano Y., Ogasawara J., Makino K., Kashima T., Ihara K., Yoshimoto A., Morikawa S., Yagishita S., Tanaka K.F. (2023). Mesolimbic dopamine release precedes actively sought aversive stimuli in mice. Nat. Commun..

[79] Gogolla N., Takesian A.E., Feng G., Fagiolini M., Hensch T.K. (2014). Sensory integration in mouse insular cortex reflects GABA circuit maturation. Neuron.

[80] Schulz S.E., Stevenson R.A. (2019). Sensory hypersensitivity predicts repetitive behaviours in autistic and typically-developing children. Autism.

[81] Wang L., Almeida L.E., Nettleton M., Khaibullina A., Albani S., Kamimura S., Nouraie M., Quezado Z.M. (2016). Altered nocifensive behavior in animal models of autism spectrum disorder: The role of the nicotinic cholinergic system. Neuropharmacology.

[82] Wolff J.J., Swanson M.R., Elison J.T., Gerig G., Pruett J.R., Styner M.A., Vachet C., Botteron K.N., Dager S.R., Estes A.M. (2017). Neural circuitry at age 6 months associated with later repetitive behavior and sensory responsiveness in autism. Mol. Autism.

[83] Monday H.R., Wang H.C., Feldman D.E. (2023). Circuit-level theories for sensory dysfunction in autism: Convergence across mouse models. Front. Neurol..

[84] Chen Y.H., Rodgers J., McConachie H. (2009). Restricted and repetitive behaviours, sensory processing and cognitive style in children with autism spectrum disorders. J. Autism Dev. Disord..

[85] Gabriels R.L., Agnew J.A., Miller L.J., Gralla J., Pan Z., Goldson E., Ledbetter J.C., Dinkins J.P., Hooks E. (2008). Is there a relationship between restricted, repetitive, stereotyped behaviors and interests and abnormal sensory response in children with autism spectrum disorders?. Res. Autism Spectr. Disord..

[86] Manor-Binyamini I., Schreiber-Divon M. (2019). Repetitive behaviors: Listening to the voice of people with high-functioning autism spectrum disorder. Res. Autism Spectr. Disord..

[87] Wang L., Almeida L.E., Spornick N.A., Kenyon N., Kamimura S., Khaibullina A., Nouraie M., Quezado Z.M. (2015). Modulation of social deficits and repetitive behaviors in a mouse model of autism: The role of the nicotinic cholinergic system. Psychopharmacology.

[88] Gandhi T., Lee C.C. (2021). Neural mechanisms underlying repetitive behaviors in rodent models of autism spectrum disorders. Front. Cell. Neurosci..

[89] Muehlmann A.M., Maletz S., King M.A., Lewis M.H. (2020). Pharmacological targeting of striatal indirect pathway neurons improves subthalamic nucleus dysfunction and reduces repetitive behaviors in C58 mice. Behav. Brain Res..

[90] Wilkes B.J., Bass C., Korah H., Febo M., Lewis M.H. (2020). Volumetric magnetic resonance and diffusion tensor imaging of C58/J mice: Neural correlates of repetitive behavior. Brain Imaging Behav..

[91] Langen M., Kas M.J., Staal W.G., van Engeland H., Durston S. (2011). The neurobiology of repetitive behavior: Of mice…. Neurosci. Biobehav. Rev..

[92] Reig R., Silberberg G. (2014). Multisensory integration in the mouse striatum. Neuron.

[93] Chang A.D., Berges V.A., Chung S.J., Fridman G.Y., Baraban J.M., Reti I.M. (2016). High-frequency stimulation at the subthalamic nucleus suppresses excessive self-grooming in autism-like mouse models. Neuropsychopharmacology.

[94] Bellistri E., Aguilar J., Brotons-Mas J.R., Foffani G., De la Prida L.M. (2013). Basic properties of somatosensory-evoked responses in the dorsal hippocampus of the rat. J. Physiol..

[95] Ferguson A.V., Latchford K.J., Samson W.K. (2008). The paraventricular nucleus of the hypothalamus–a potential target for integrative treatment of autonomic dysfunction. Expert Opin. Ther. Targets.

[96] Salay L.D., Ishiko N., Huberman A.D. (2018). A midline thalamic circuit determines reactions to visual threat. Nature.

[97] Chen C.H., Hu J.M., Zhang S.Y., Xiang X.J., Chen S.Q., Ding S.L. (2021). Rodent area prostriata converges multimodal hierarchical inputs and projects to the structures important for visuomotor behaviors. Front. Neurosci..

[98] Mikellidou K., Kurzawski J.W., Frijia F., Montanaro D., Greco V., Burr D.C., Morrone M.C. (2017). Area prostriata in the human brain. Curr. Biol..

[99] Gelfo F., Mandolesi L., Serra L., Sorrentino G., Caltagirone C. (2018). The neuroprotective effects of experience on cognitive functions: Evidence from animal studies on the neurobiological bases of brain reserve. Neuroscience.

[100] Nithianantharajah J., Hannan A.J. (2006). Enriched environments, experience-dependent plasticity and disorders of the nervous system. Nat. Rev. Neurosci..

[101] Begenisic T., Spolidoro M., Braschi C., Baroncelli L., Milanese M., Pietra G., Fabbri M.E., Bonanno G., Cionni G., Maffei L. (2011). Environmental enrichment decreases GABAergic inhibition and improves cognitive abilities, synaptic plasticity, and visual functions in a mouse model of Down syndrome. Front. Cell Neurosci..

[102] Engineer N.D., Percaccio C.R., Pandya P.K., Moucha R., Rathbun D.L., Kilgard M.P. (2004). Environmental enrichment improves response strength, threshold, selectivity, and latency of auditory cortex neurons. J. Neurophysiol..

[103] Huang Y., Jiang H., Zheng Q., Fok A.H.K., Li X., Lau C.G., Lai C.S.W. (2021). Environmental enrichment or selective activation of parvalbumin-expressing interneurons ameliorates synaptic and behavioral deficits in animal models with schizophrenia-like behaviors during adolescence. Mol. Psychiatry.

[104] Gabriel A.F., Paoletti G., Della Seta D., Panelli R., Marcus M.A., Farabollini F., Giancarlo C., Joosten E.A. (2010). Enriched environment and the recovery from inflammatory pain: Social versus physical aspects and their interaction. Behav. Brain Res..

[105] Schneider T., Turczak J., Przewłocki R. (2006). Environmental enrichment reverses behavioral alterations in rats prenatally exposed to valproic acid: Issues for a therapeutic approach in autism. Neuropsychopharmacology.

[106] Vachon P., Millecamps M., Low L., Thompsosn S.J., Pailleux F., Beaudry F., Bushnell C.M., Stone L.S. (2013). Alleviation of chronic neuropathic pain by environmental enrichment in mice well after the establishment of chronic pain. Behav. Brain Funct..

[107] Clipperton-Allen A.E., Zhang A., Cohen O.S., Page D.T. (2021). Environmental enrichment rescues social behavioral deficits and synaptic abnormalities in pten haploinsufficient mice. Genes.

