# Supplementary material for: Environmental Enrichment Attenuates Repetitive Behavior and Alters the Functional Connectivity of Pain and Sensory Pathways in C58 Mice
Source: Cells. 2024 Nov 21;13(23):1933. doi: 10.3390/cells13231933 (PMC11640393; doi:10.3390/cells13231933)
Supplement: Supplementary file 1 [file cells-13-01933-s001.zip › cells-3298113-supplementary.pdf]

Supplementary Table S1. Resting state networks resulting from an independent component analysis of the older mouse cohort. Region indicates the brain region(s) with the highest z-scores.

Network Name (Component)	Hemisphere	Region	Z Score
Visual network (1)	Left	Primary visual cortex	10.96
Somatosensory network (2)	Left	Primary somatosensory cortex	12.78
Somatosensory network (3)	Right	Primary somatosensory cortex	13.44
Visual network (4)	Right	Postsubiculum	14.34
Superior colliculus/anterior pretectal nucleus network (5)	Left	Superior colliculus	6.66
		Anterior pretectal nucleus	6.65
Amygdala/piriform/motor network (6)	Right	Lateral amygdala nucleus	6.47
Default mode-like network (7)	Both	Corpus callosum body	13.98
		Retrosplenial cortex	13.82
		Anterior cingulate cortex	13.51
Trigeminal nerve network (8)	Left	Principal sensory nucleus of the trigeminal nerve	9.29
Striatal network (9)	Left	Striatum	23.72
Cerebellar network (10)	Left	Left lobule III	10.91
Motor network (11)	Right	Primary motor cortex	14.70
Striatal network (12)	Right	Striatum	18.81
Rostral limbic network (13)	Both	Taenia tecta	21.69
Cerebellar network (14)	Right	Right interposed nucleus	11.66
Thalamic/hypothalamic network (15)	Both	Central medial thalamic nucleus	17.39
Reticular formation network (16)	Left	Tegmental reticular nucleus	23.76
Motor network (17)	Left	Secondary motor cortex	15.71
Reticular formation network (18)	Right	Pontine reticular nucleus	17.77
Caudal striatal network (19)	Both	Right ventral caudal striatum	11.64
Brainstem network (20)	Both	Gigantocellular reticular nucleus	13.76

Supplementary Table S2. Resting state networks resulting from an independent component analysis of the younger mouse cohort. Region indicates the brain region(s) with the highest z-scores.

Network Name (Component)	Hemisphere	Region	Z Score
Visual network (1)	Left	Primary visual cortex	10.60
Somatosensory network (2)	Left	Primary somatosensory cortex	9.38
Visual network (3)	Right	Primary visual cortex	11.76
Sensorimotor network (4)	Left	Primary motor cortex	13.80
Somatosensory network (5)	Right	Supplemental somatosensory area	15.07
Striatal network (6)	Left	Ventral striatum	15.99
Pain/sensory network (7)	Left	Posterior thalamic complex	8.71
Caudal striatal network (8)	Left	Ventrolateral striatum	9.24
Cerebellar/brainstem network (9)	Left	Lobule III	11.37
Prefrontal cortex network (10)	Both	Infralimbic cortex	18.31
		Prelimbic cortex	18.31
Hippocampal/motor/limbic network (11)	Right	Hippocampus CA1	6.33
Reticular formation network (12)	Left	Pontine reticular nucleus	9.22
Striatal network (13)	Right	Ventral striatum	15.44
Sensorimotor network (14)	Right	Primary somatosensory cortex	11.97
Cerebellar/brainstem network (15)	Right	Parabrachial nucleus	8.61
		Lobule II	8.61
Subiculum network (16)	Right	Subiculum	10.78
Default mode-like network (17)	Both	Corpus callosum	8.22
		Anterior cingulate cortex	8.21
Reticular formation network (18)	Right	Pontine reticular nucleus	20.26
Thalamus/hypothalamus network (19)	Both	Central medial thalamic nucleus	9.42
Brainstem network (20)	Both	Gigantocellular reticular nucleus	8.11

Supplementary Table S3. Resting State Networks (ICA Components) and brain regions with significant (FWE-corrected p<0.05) functional connectivity differences between mouse strains in the older 6-week post-weaning cohort. L= left. R=right. L/R = Left and right**.**

Network (Component)	Brain Area	Brain Region	Strain Difference	Corrected p-value
L Somatosensory network (2)	Cortex	L Hippocampus dentate gyrus	C58 > C57	< 0.006
		L Hippocampus CA1	C58 > C57	< 0.008
		L Hippocampus CA3	C58 > C57	< 0.02
		L Dorsal auditory area	C58 > C57	< 0.02
		L Posterior auditory area	C58 > C57	< 0.02
L Superior colliculus/anterior pretectal nucleus network (5)	Cortex	R Anterior cingulate area	C58 > C57	< 0.04
		L Claustrum	C58 > C57	< 0.05
		R Endopiriform nucleus	C58 > C57	< 0.04
		L Hippocampus CA1	C58 > C57	< 0.007
		L/R Hippocampus CA3	C58 > C57	< 0.02
		L Hippocampus dentate gyrus	C58 > C57	< 0.02
		L Primary motor area	C58 > C57	< 0.02
		L Primary somatosensory area	C58 > C57	< 0.008
		L Supplemental somatosensory area	C58 > C57	< 0.01
		R Piriform area	C58 > C57	< 0.03
		L Parasubiculum	C58 > C57	< 0.02
		L Presubiculum	C58 > C57	< 0.02
		L/R Retrosplenial area	C58 > C57	< 0.02
	Basal Forebrain	R Olfactory tubercle	C58 > C57	< 0.03
		L Zona incerta	C58 > C57	< 0.02
	Basal Ganglia	R Nucleus accumbens	C58 > C57	< 0.05
		R Substantia nigra pars compacta	C58 > C57	< 0.02
		R Substantia nigra pars reticulata	C58 > C57	< 0.02
	Thalamus	R Central medial nucleus	C58 > C57	< 0.05
		L Ethmoid nucleus	C58 > C57	< 0.02
		R Interanterodorsal nucleus	C58 > C57	< 0.03
		L Intermediodorsal nucleus	C58 > C57	< 0.05
		L Medial geniculate complex	C58 > C57	< 0.02
		L Mediodorsal nucleus	C58 > C57	< 0.04
		Parataenial nucleus	C58 > C57	< 0.03
		R Paraventricular nucleus	C58 > C57	< 0.04
		L Posterior triangular nucleus	C58 > C57	< 0.03
		R Subparafascicular nucleus	C58 > C57	< 0.03
	Hypothalamus	Unspecified	C58 > C57	< 0.04

	Midbrain	L Interstitial nucleus of Cajal	C58 > C57	< 0.02
		L/R Midbrain reticular nucleus	C58 > C57	< 0.02
		R Pedunculopontine nucleus	C58 > C57	< 0.03
		L Periaqueductal gray	C58 > C57	< 0.02
		L/R Red nucleus	C58 > C57	< 0.02/<0.03
		R Superior colliculus	C58 > C57	< 0.03
	Cerebellum	L Crus I	C58 > C57	< 0.04
		L Crus II	C58 > C58	< 0.04
		L/R Declive	C58 > C57	< 0.05/ < 0.04
		L Flocculus	C58 > C57	< 0.04
		L Paraflocculus	C58 > C57	< 0.03
		L/R Lobules IV-V	C58 > C57	< 0.02/ < 0.05
		L Simple Lobule	C58 > C57	< 0.02
Default mode-like network (7)	Cortex	L Primary motor area	C58 < C57	< 0.03
		R Secondary motor area	C58 < C57	< 0.01
		L Primary somatosensory area	C58 < C57	< 0.04
		L/R Retrosplenial area	C58 < C57	< 0.005
L Striatal network (9)	Cortex	L Anterior cingulate	C58 > C57	< 0.05
		R Infralimbic area	C58 > C57	< 0.05
		R Orbitofrontal area	C58 > C57	< 0.05
		L Primary somatosensory area	C58 > C57	< 0.006
		R Taenia tecta	C58 > C57	< 0.05
	Midbrain	L Inferior colliculus	C58 > C57	< 0.04
		L Periaqueductal gray	C58 > C57	< 0.05
	Cerebellum	L Simple lobule	C58 > C57	< 0.03
R Striatal network (12)	Basal forebrain	L Zona incerta	C58 > C57	< 0.03
	Thalamus	L Ventral posterolateral nucleus	C58 > C57	< 0.04
		L Ventral posteromedial	C58 > C57	< 0.03
		L Reticular nucleus	C58 > C57	< 0.04

L Cerebellar network (10)	Midbrain	R Periaqueductal gray	C58 > C57	< 0.02
Rostral limbic network (13)	Cortex	R Lateral entorhinal area	C58 < C57	< 0.01
L Motor network (17)	Cortex	L Lateral visual area	C58 < C57	< 0.03
		L Laterointermediate area	C58 < C57	< 0.04
		L Primary visual area	C58 < C57	< 0.04
Caudal striatal network (19)	Cortex	R Primary auditory area	C58 > C57	< 0.006
		R. Dorsal auditory area	C58 > C57	< 0.006
		L Claustrum	C58 > C57	< 0.006
		R Ectorhinal area	C58 > C57	< 0.05
		L Cortical amygdalar area	C58 > C57	< 0.0
		L/R Endopiriform nucleus	C58 > C57	< 0.006/< 0.003
		L/R Piriform area	C58 > C57	< 0.007/ < 0.03
		R Lateral entorhinal area	C58 > C57	< 0.05
		R Medial entorhinal area	C58 > C57	< 0.02
		L Gustatory area	C58 > C57	< 0.006
		R Hippocampus CA1 field	C58 > C57	< 0.007
		R Hippocampus CA3 field	C58 > C57	< 0.004
		R Hippocampus dentate gyrus	C58 > C57	< 0.006
		L Agranular insular area	C58 > C57	< 0.007
		R Primary visual area	C58 > C57	< 0.02
		R Laterointermediate area	C58 > C57	< 0.02
		R Lateral visual area	C58 > C57	< 0.03
		R Posterolateral visual area	C58 > C57	< 0.02
		L/R Primary somatosensory area	C58 > C57	< 0.006/ < 0.02
		R Supplemental somatosensory area	C58 > C57	< 0.01
		R Subiculum	C58 > C57	< 0.02
		R Parasubiculum	C58 > C57	< 0.02
		R Prosubiculum	C58 > C57	< 0.02
	Amygdala	L/R Basolateral nucleus	C58 > C57	< 0.05/ < 0.002
		R Basomedial nucleus	C58 > C57	< 0.002

		L/R Central nucleus	C58 > C57	< 0.01/ < 0.005
		R Lateral nucleus	C58 > C57	< 0.005
		R Medial nucleus	C58 > C57	< 0.002
		R Posterior nucleus	C58 > C57	< 0.005
	Basal ganglia	L Ventrolateral Striatum	C58 > C57	< 0.006
		R Substantia nigra pars reticulata	C58 > C57	< 0.009
	Thalamus	Nucleus reuniens	C58 > C57	< 0.05
	Hypothalamus	R Dorsal nucleus	C58 > C57	< 0.04
	Midbrain	L Inferior colliculus central nucleus	C58 > C57	< 0.04
		L inferior colliculus external nucleus	C58 > C57	< 0.04
	Cerebellum	L Lobule III	C58 > C57	< 0.02
		L Lobules IV-V	C58 > C57	< 0.02
Brainstem/gigantocellular reticular network (20)				
	Cortex	R Anterior olfactory nucleus	C58 > C57	< 0.02
		R Olfactory areas	C58 > C57	< 0.02
		R Taenia tecta dorsal part	C58 > C57	< 0.02
	Hypothalamus	R Anterior nucleus	C58 > C57	< 0.04
		R Dorsomedial nucleus	C58 > C57	< 0.02
		R Lateral hypothalamic area	C58 > C57	< 0.02
		R Paraventricular nucleus	C58 > C57	< 0.02
		R Perifornical	C58 > C57	< 0.02
		R Periventricular	C58 > C57	< 0.04
		R Retrochiasmic	C58 > C57	< 0.04
		R Subparaventricular zone	C58 > C57	< 0.04
		Suprachiasmic nucleus	C58 > C57	< 0.03
	Midbrain	Nucleus raphe pontis	C58 > C57	< 0.04

Supplementary Table S4. Resting state networks (ICA components) with significant functional connectivity differences (FWE-corrected p<0.05) correlated with repetitive motor scores in C58 and C57 mice at 6 weeks post-weaning.

Network (Component)	Brain Area	Brain Region	Correlation	Corrected p-value
Right somatosensory network (3)	Basal ganglia	R Striatum	positive	< 0.03
	Thalamus	R Lateral dorsal nucleus	positive	< 0.02
		R Posterior complex	positive	< 0.03
		R Reticular nucleus	positive	< 0.02
		R Ventral posteromedial nucleus	positive	< 0.03
		R Ventral posterolateral nucleus	positive	< 0.03
Caudal striatal network (19)	Amygdala	R medial nucleus	positive	< 0.05
		R posterior nucleus	positive	< 0.05
	White matter tracts	Alveus	positive	< 0.05
		Internal capsule	positive	< 0.05
		Optic tract	positive	< 0.05

Supplementary Table S5. Resting State Networks (ICA Components) and brain regions with significant (FWE-corrected p<0.05) functional connectivity differences between housing conditions in the older 6-week post-weaning cohort. Both mouse strains included. EE= environmental enrichment. SH= standard housed. L= left. R=right. L/R = Left and right.

Network (Component)	Brain Area	Brain Region	Housing Difference	Corrected p-value
Left Somatosensory Network (2)	Cortex	R Hippocampus CA1	EE > SH	< 0.02
		R Hippocampus dentate gyrus molecular layer	EE > SH	< 0.01
		R Hippocampus dentate gyrus granule cell layer	EE > SH	< 0.02
		R Prosubiculum	EE > SH	< 0.02
	Midbrain	R Anterior pretectal nucleus	EE > SH	< 0.01
		R Nucleus of the optic tract	EE > SH	< 0.02
		R Olivary pretectal nucleus	EE > SH	< 0.02
		R Posterior pretectal nucleus	EE > SH	< 0.03

Supplementary Table S6. Resting State Networks (ICA Components) and brain regions with nearly significant (FWE-corrected p<0.10) functional connectivity differences between housing conditions in the older 6-week post-weaning cohort. Both mouse strains included. EE= environmental enrichment. SH= standard housed. L= left. R=right. L/R = Left and right.

Network (Component)	Brain Area	Brain Region	Housing Difference	Corrected p-value
Caudal striatal network (19)	Thalamus	R Perireunensis nucleus	EE < SH	< 0.10
		R Reuniens nucleus	EE < SH	< 0.09
		R Xiphoid nucleus	EE < SH	< 0.09
	Hypothalamus	R Paraventricular nucleus	EE < SH	< 0.10

Supplementary Table S7. Resting State Networks (ICA Components) and brain regions with significant (FWE-corrected p<0.05) functional connectivity differences between housing conditions in C58 mice at 3 weeks post-weaning. EE= environmental enrichment. SH= standard housed. L= left. R=right.

Network (Component)	Brain Area	Brain Region	Housing Difference	Corrected p-value
Left visual network (1)	Cortex	R Area prostriata	EE < SH	< 0.007
		R Retrosplenial cortex	EE < SH	< 0.02
	Hippocampus	R Parasubiculum	EE < SH	< 0.04
		R Presubiculum	EE < SH	< 0.02

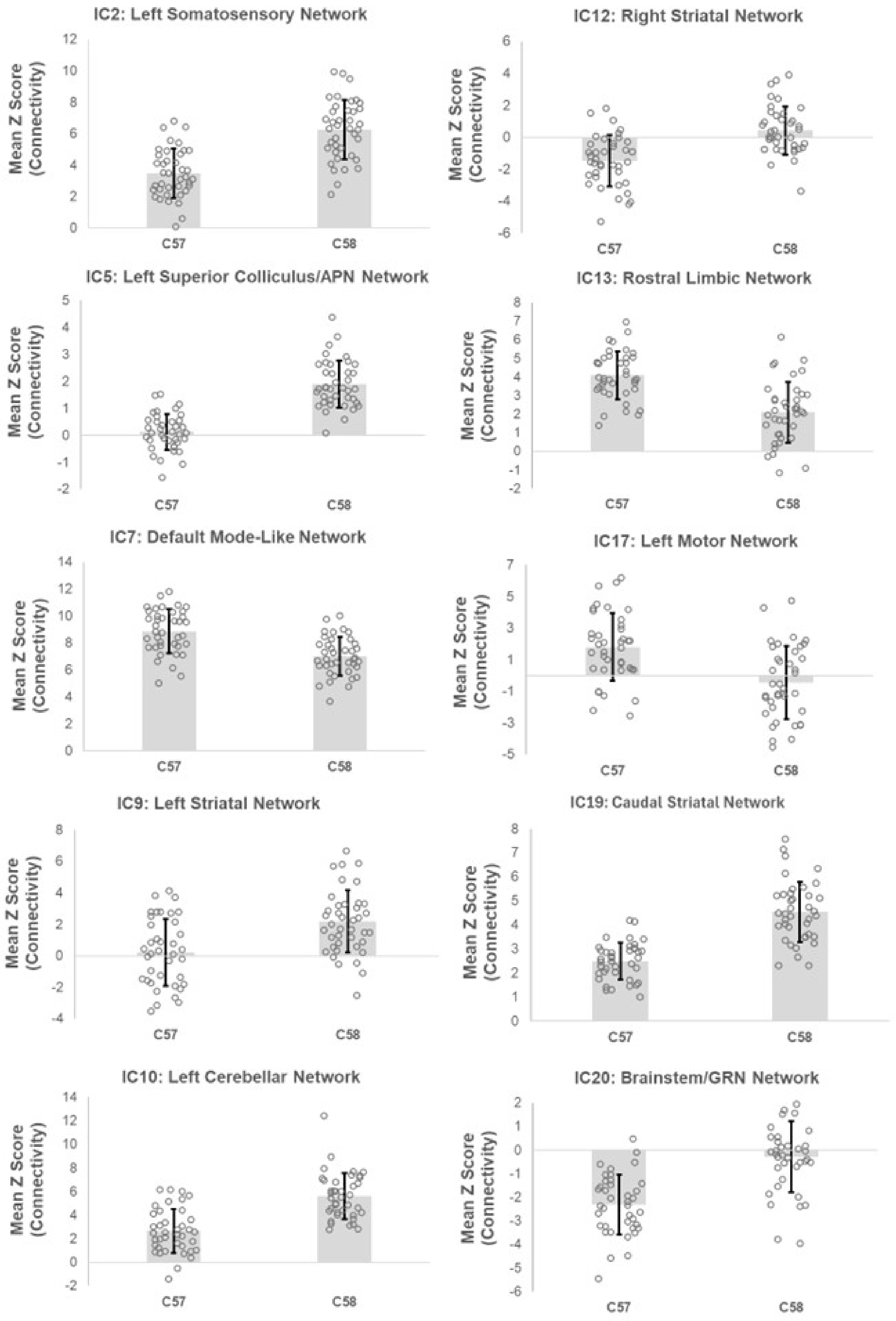

IC2: Left Somatosensory Network
Mean Z Score (Connectivity)
C57
C58
IC12: Right Striatal Network
Mean Z Score (Connectivity)
C57
C58
IC5: Left Superior Colliculus/APN Network
Mean Z Score (Connectivity)
C57
C58
IC13: Rostral Limbic Network
Mean Z Score (Connectivity)
C57
C58
IC7: Default Mode-Like Network
Mean Z Score (Connectivity)
C57
C58
IC17: Left Motor Network
Mean Z Score (Connectivity)
C57
C58
IC9: Left Striatal Network
Mean Z Score (Connectivity)
C57
C58
IC19: Caudal Striatal Network
Mean Z Score (Connectivity)
C57
C58
IC10: Left Cerebellar Network
Mean Z Score (Connectivity)
C57
C58
IC20: Brainstem/GRN Network
Mean Z Score (Connectivity)
C57
C58

Figure S1. Mean z-values for significant network differences between mouse strains. Z values reflect the functional connectivity strength between the network and a mask of significant brain regions (FWE-corrected p<0.05) identified by a t-test conducted using FSL dual regression. Data points represent Z values extracted from individual subject z-maps.

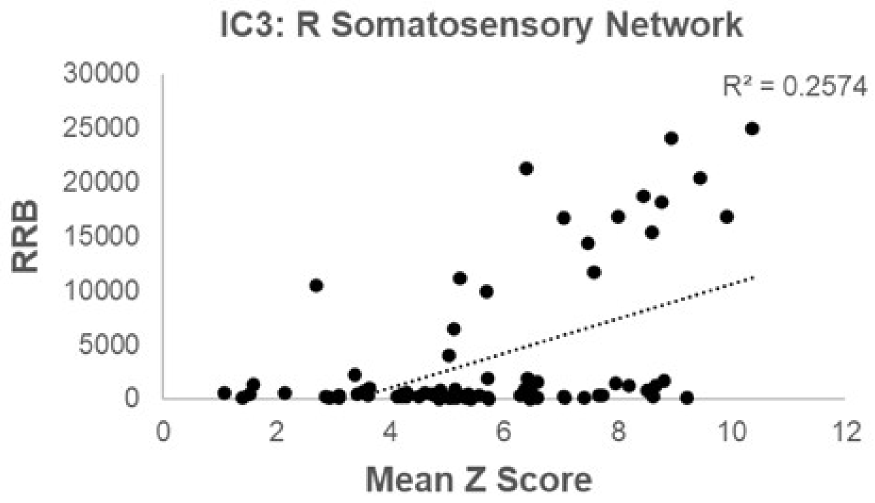

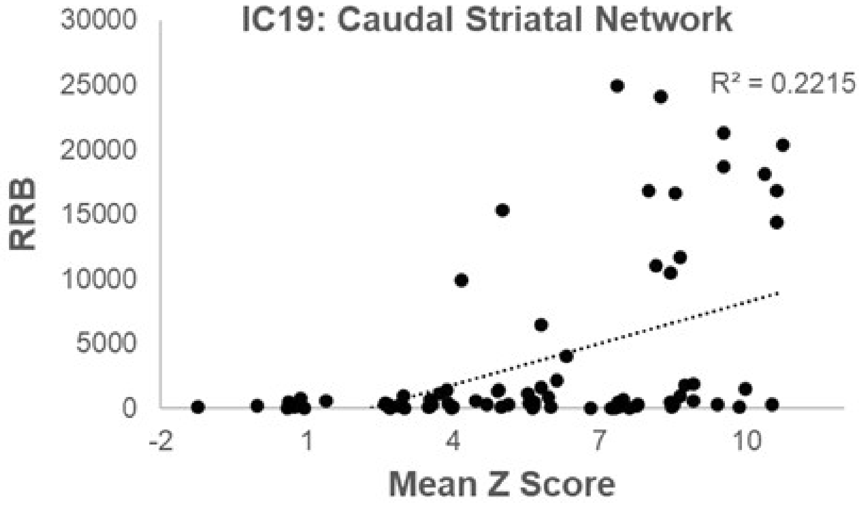

Figure S2. Correlation between subject Z values and repetitive behavior (RRB) scores for functional networks with significant (FWE-corrected p<0.05) RRB associations in the older 6-week post-weaning cohort. Z values reflect the functional connectivity strength between the network and a mask of significant brain regions identified by a correlation analysis conducted using FSL dual regression. Z values extracted from individual subject z-maps.

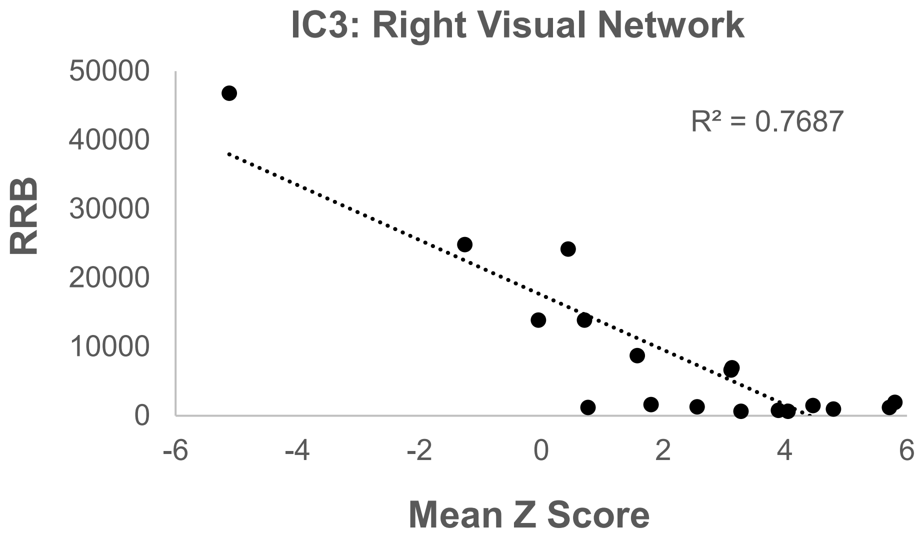

Figure S3. Correlation between subject Z values and repetitive behavior (RRB) scores for functional networks with significant (FWE-corrected $p<0.05$) RRB associations in the younger 3-week post-weaning cohort. Z values reflect the functional connectivity strength between the network and a mask of significant brain regions identified by a correlation analysis conducted using FSL dual regression. Z values extracted from individual subject z-maps.

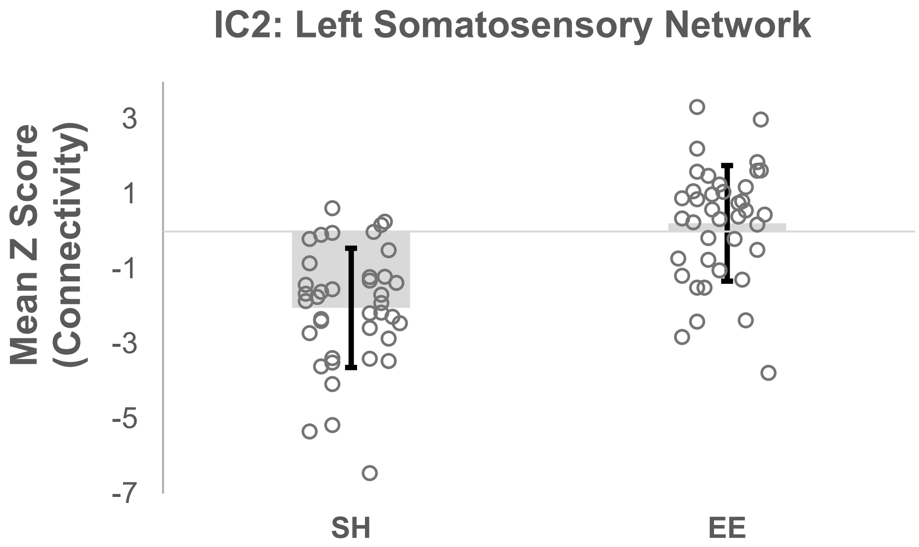

Figure S4. Mean z-values for significant network differences between housing conditions in the older 6-week post-weaning cohort. Z values reflect the functional connectivity strength between the network and a mask of significant brain regions (FWE-corrected $p<0.05$) identified by a t-test conducted using FSL dual

regression. Data points represent Z values extracted from individual subject z-maps. SH = Standard housing. EE = Environmental enrichment.

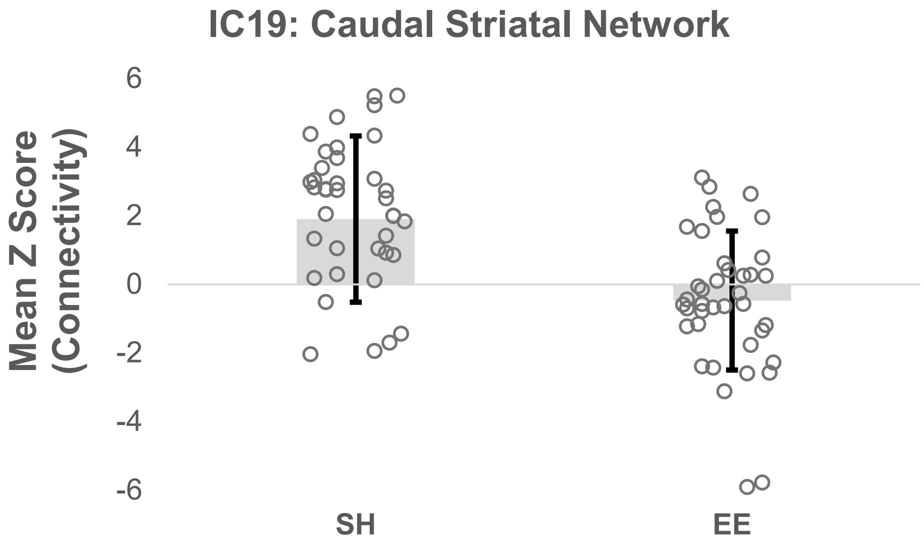

Figure S5. Mean z-values for nearly significant network differences between housing conditions in the older 6-week post-weaning cohort. Z values reflect the functional connectivity strength between the network and a mask of nearly significant brain regions (FWE-corrected p<0.10) identified by a t-test conducted using FSL dual regression. Data points represent Z values extracted from individual subject z-maps. SH = Standard housing. EE = Environmental enrichment.

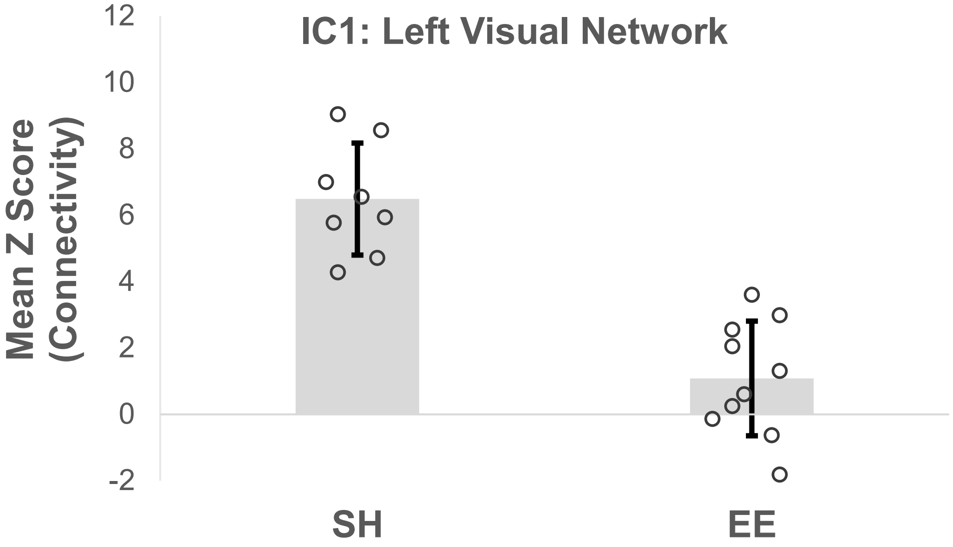

Figure S6. Mean z-values for significant network differences between housing conditions in the younger 3-week post-weaning cohort. Z values reflect the functional connectivity strength between the network and a mask of significant brain regions (FWE-corrected p<0.05) identified by a t-test conducted using FSL dual regression. Data points represent Z values extracted from individual subject z-maps. SH = Standard housing. EE = Environmental enrichment.
